# Investigation of Antitumor Activity of Modified Citrus Pectin: Oral and Intravenous Administration Assessed via Molecular Imaging

**DOI:** 10.1021/acs.biomac.5c00915

**Published:** 2026-03-24

**Authors:** Fábio Fernando Alves da Silva, Sofia Nascimento dos Santos, Lucas de Freitas Pedrosa, Vinicius Gonçalves Rodrigues, Jonathas Xavier Pereira, Jhonatas Pedrosa Marim Pereira, Dino Seigo Gushiken Junior, Thiécla Katiane Osvaldt Rosales, Luís Alberto Pereira Dias, Patrick Jack Spencer, João Paulo Fabi, Emerson Soares Bernardes

**Affiliations:** † Centro de Radiofarmácia, Comissão Nacional de Energia Nuclear, 119500 Instituto de Pesquisas Energéticas e Nucleares, São Paulo, São Paulo 05508-000, Brasil; ‡ School of Biomedical Engineering and Imaging Sciences, King’s College London, London SE1 7EH, U.K.; § Department of Food Science and Experimental Nutrition, School of Pharmaceutical Sciences, University of São Paulo, São Paulo, São Paulo 05508-900, Brazil; ∥ Instituto de Patologia Tropical e Saúde Pública (IPTSP), Universidade Federal de Goiás (UFG), Goiânia, Goiás 74605-050, Brasil; ⊥ Centro de Biotecnologia, Comissão Nacional de Energia Nuclear, Instituto de Pesquisas Energéticas e Nucleares, São Paulo, São Paulo 05508-000, Brasil

## Abstract

This study employed molecular imaging to evaluate MCP (PectaSol-C, modified citrus pectin, a complex polysaccharide with antitumor potential) absorption and pharmacokinetics following oral and intravenous (IV) administration. MCP was radiolabeled with technetium-99m (^99m^Tc) ([^99m^Tc]­MCP), allowing precise in vivo tracking. Imaging and biodistribution analyzes revealed low tumor uptake of IV [^99m^Tc]­MCP, with predominant renal and hepatobiliary clearance. Within tumors, MCP was detected at low levels and did not bind to viable cells. Consistent with these findings, IV administration produced only modest antitumor effects (∼50% tumor growth reduction) in SKOV-3 (ovarian), MKN45 (gastric), and 4T1 (breast) grafts, whereas oral administration was ineffective due to extremely poor absorption (bioavailability <0.01%). Notably, faster clearance of [^99m^Tc]­MCP in galectin-3 (Gal-3) knockout mice suggests a role for Gal-3 in systemic retention or an indirect contribution to antitumor activity. These findings provide new insights into MCP pharmacological profile, highlight the limitations of oral delivery, and underscore the need for improved delivery strategies to enhance the therapeutic potential of pectin-based cancer treatments.

## Graphical abstract

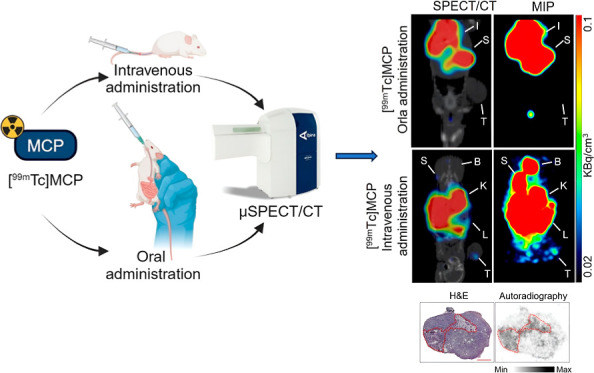

## 1 Introduction

Cancer remains a major global health burden and is among the leading causes of mortality, with an estimated 9.7 million deaths reported in 2022.^1,2^ In the United States, approximately 1.9 million new cancer cases and 609,360 deaths were projected for 2022, while in Brazil, 625,000 new cases are anticipated, potentially rising to 704,000 by 2025.^3–5^ Given this persistent and escalating impact, the pursuit of novel therapeutic options to improve treatment efficacy and patient outcomes remains a high priority.

Modified citrus pectin (MCP) is a structurally complex heteropolysaccharide derived from citrus pectin through controlled reduction of its molecular weight. It is primarily composed of galacturonic acid residues arranged into distinct polysaccharide domains—homogalacturonan (HG), rhamnogalacturonan-I (RG-I), and substituted galacturonans—along with other monosaccharide components.^6–9^ In multiple in vitro cancer models, MCP has been shown to inhibit cell proliferation, modulate immune responses, reduce metastasis, and overcome chemoresistance.^10–14^ Some in vitro data suggest that MCP may bind and inhibit galectin-3 (Gal-3), a lectin implicated in tumor progression.^15–18^ However, because Gal-3 belongs to a larger galectin family with broad β-galactoside specificity, it remains difficult to confirm precisely which galectins MCP might be blocking in vivo, and no direct in vivo evidence has yet demonstrated Gal-3 inhibition as the principal mechanism of action.

PectaSol-C is a commercial formulation of MCP derived from water-soluble citrus pectin through pH/temperature modification, and it is used as a soluble dietary supplement.^19^ This specific brand of MCP contains β-galactosides and has been employed in *in vitro* studies of cancer as a potential adjunct therapy.^12,13,20,21^ Most preclinical and clinical investigations with MC*P* formulations (including PectaSol-C) have been conducted via oral administration. For example, oral MCP has shown promising activity in *in vivo* models of melanoma,^22^ breast and colon carcinoma,^23^ bladder cancer,^18^ colorectal cancer,^24^ prostate cancer,^25^ breast cancer,^14^ and thyroid carcinoma.^26^ A caveat is that a 20% MCP diet over 21 days produced negative outcomes in a colorectal cancer model.^27^ In a Phase II clinical study, oral administration of PectaSol increased the prostate-specific antigen doubling time in 80% of men with biochemical relapse of prostate cancer over six months (NCT01681823).^28–30^

Oral administration remains the prevailing route in clinical trials largely due to its noninvasiveness and established safety, despite a lack of quantitative pharmacokinetic data confirming systemic bioavailability or effective tumor exposure. However, whether MCP achieves sufficient systemic exposure, particularly with oral dosing, remains uncertain. To resolve this quantitative gap, we sought a whole-body method to measure MCP absorption, biodistribution, and clearance in vivo. Accordingly, we selected SPECT because it enables sensitive, quantitative, whole-body, serial imaging of tracer biodistribution in small animals and is widely available in preclinical facilities, facilitating reproducibility and future translation. Technetium-99m (^99m^Tc) was chosen because its physical properties are well aligned with the pharmacokinetic window of MCP: a 6 h physical half-life (*t*_1/2_ = 6 h) supports imaging over several hours with low dose, and a 140 keV γ emission is optimal for γ-camera/SPECT detection. ^99m^Tc is inexpensive, produced on-site from a ^99^Mo/^99m^Tc generator, and has a long regulatory track record across radiopharmaceutical classes. Importantly for polysaccharides, ^99m^Tc can be incorporated by reduction and complexation without introducing bulky chelators, preserving MCP’s native characteristics and enabling high radiochemical yields. Together, SPECT with ^99m^Tc provides a practical, sensitive platform to quantify MCP absorption, tissue distribution, and clearance in vivo.^31–37^

While oral MCP is well studied, alternative routes of administration (e.g., intravenous, intraperitoneal, intratumoral) remain less explored. Notably, no published studies have used the PectaSol-C formulation via the intravenous route. Nonetheless, other MCP formulations (e.g., GCS-100, GM-CT-01) have been evaluated intravenously in both preclinical and clinical settings, demonstrating reduced metastasis in animal models^10,12–14,18,20,21,38–40^ and potential kidney protection in chronic kidney disease patients (NCT02333955, NCT01717248).

In this study, we address several open questions regarding MCP’s mechanism of action, pharmacokinetics, and biodistribution, particularly in light of the in vitro studies suggesting Gal-3 binding. This study presents the successful radiolabeling of MCP (specifically, PectaSol-C) with technetium-99m (^99m^Tc), enabling the tracking of its in vivo absorption, distribution, and elimination in a tumor in vivo model. By comparing intravenous and oral routes, we sought to determine whether MCP reaches the tumor effectively, evaluate any resulting antitumor activity, and gain insights into whether Gal-3 or other galectins might be involved in MCP’s biological effects in vivo. Through a combination of molecular imaging, biochemical analysis, and histopathological examination, our findings aim to clarify how MCP exerts its antitumor activity and to underscore the potential importance of different administration routes.

## 2 Materials and Methods

### 2.1 Materials

MCP was PectaSol-C (ecoNugenics). The product was used as received and stored per the supplier’s instructions. Supplier documents indicate a water-soluble, modified citrus pectin with reduced molecular weight and degree of esterification; monosaccharide composition includes galacturonic acid with neutral sugars (e.g., galactose, arabinose, rhamnose). MCP (PectaSol-C) was utilized in subcutaneous tumor inoculation, in vivo tumor assays, radiolabeling, bioavailability, biodistribution studies, and pharmacokinetic analyzes. Biochemical assays employed reagent kits including Transaminase AST (TGO) (aspartate aminotransferase/oxalacetic transaminase) kinetic test (Bioclin KO48-9), Creatinine Automated kinetic test (Bioclin), Alkaline Phosphatase (ALP) IFCC kinetic test (Bioclin), Transaminase ALT (alanine aminotransferase) kinetic test (Bioclin KO49-6), Urea UV test (Bioclin K056-1), and Glucose monoreagent enzymatic reagent (Bioclin KO82). ITLC-SG plates (Agilent Technologies, CA, USA) were used in the quality control and stability assays of radiolabeled MCP ([^99m^Tc]­MCP), enabling the quantification of radiochemical purity and identification of radiolabeling products. MWCO Amicon Ultra-4 Centrifugal Filters (Millipore) were employed to produce MCP fractions with different molecular weights, which were subsequently analyzed in hemagglutination assays, biodistribution studies, and other functional analyzes.

Neutral sugars (l-arabinose, d-galactose, d-glucose, d-fucose, d-mannose, l-rhamnose, and d-xylose) and uronic acids (d-glucuronic and D-galacturonic acids), obtained from Sigma (St. Louis, MO, USA), were utilized for polysaccharide composition analysis via high-performance anion-exchange chromatography with pulsed amperometric detection (HPAEC-PAD) after hydrolysis of MCP fractions. The Novex NuPAGE SDS-PAGE gel system (Invitrogen), Full Ranger Rainbow ladder (Z892 53410), and Bio-Safe Coomassie blue stain (1610786) were used to assess protein content and molecular weight in SDS-PAGE experiments. A Sepharose/Gal-3 column (Cyanogen bromide-activated Sepharose CNBr C9142-5G) was used in affinity assays to evaluate the binding interaction of radiolabeled MCP ([^99m^Tc]­MCP) with Gal-3. ^99m^Tc (sodium pertechnetate) samples, provided through donations of [^99^Mo/^99m^Tc] generators from the Instituto de Pesquisas Energéticas e Nucleares (IPEN), São Paulo, Brazil, were essential for radiolabeling MCP.

### 2.2 Subcutaneous Inoculation of Tumor Cells in Mice

Subcutaneous inoculation of tumor cells was performed to establish the tumor xenograft and syngeneic models used in this study. BALB/c, BALB/c nude and C57BL/6 mice were obtained from the Bioterium of the Nuclear and Energy Research Institute (IPEN), São Paulo, Brazil. All experimental procedures were conducted in accordance with the ARRIVE guidelines and the Guidance on the Operation of the Animals (Scientific Procedures) Act 1986 and were approved by the Institutional Animal Ethics Committee (Protocol numbers DAHEICB-071, 16/22, and 2021/05). The sex of the animals did not influence the experimental results.^41,42^

Subcutaneous inoculation of tumor cells was performed on female and male BALB/c mice (syngeneic tumor model) or BALB/c nude mice (xenograft model), at 6 to 8 weeks of age, with 1 × 10^6^ SKOV-3 cells inoculated into BALB/c nude female mice, 1 × 10^6^ MKN45 cells into BALB/c nude male mice, and 1 × 10^6^ 4T1 cells into BALB/c female mice. Each group consisted of five animals. Once tumors reached approximately 0.5 cm^3^, biodistribution studies and μSPECT/CT imaging were conducted; for in vivo tumor assays, treatment was initiated when tumors reached approximately 50 mm^3^ (see below). SKOV-3 was chosen to enable comparison with prior MCP studies;^20,21^ MKN45 was included to examine a gastrointestinal carcinoma model not previously evaluated with MCP; and 4T1 cells were included to study the effects of MCP in an immunocompetent syngeneic BALB/c-4T1 tumor model.

### 2.3 In Vivo Tumor Assay

The in vivo tumor assay was conducted to evaluate the antitumor effects of MCP in tumor xenograft and syngeneic models. In this assay, BALB/c nude mice were subcutaneously injected with xenografts of SKOV-3 cells (in female mice), or MKN45 cells (in male mice), and BALB/c with 4T1 syngeneic model cells (female mice), respectively with 1 × 10^6^ cells per animal. The animals were then treated with oral or intravenous administration of MCP (PectaSol-C/Modified Citrus Pectin/ecoNugenics). When the tumors reached approximately 50 mm^3^, the animals were divided into four groups and treated daily as follows: oral administration of vehicle (PBS, 100 μL) or MCP (200 mg/kg in 100 μL), or IV administration of vehicle (PBS, 100 μL) or MCP (5–10 mg/kg in 100 μL), depending on the experiment/model as specified in the corresponding Results/figure legends) for 21 days in SKOV-3 and MKN45, or 15 days in 4T1 cells, due to its aggressiveness and faster growth rate. Each group consisted of five animals.

Dose selection and safety rationale. Oral MCP (PectaSol-C) was dosed at 200 mg/kg to offset low/variable GI (Gastrointestinal) absorption reported for MCP; IV was dosed at 10 mg/kg because bioavailability is 100%. Gavage volumes were kept within institutional guidelines. This study was not powered for formal toxicology; clinical chemistry was collected to screen tolerability and contextualize PK/PD (pharmacokinetics/pharmacodynamics).

Tumor volume was measured daily using an automatic caliper and calculated using the formula

$$tumor\;volume\;({mm}^{3})=0.5\times \left(larger\;diameter\right)\times {\left(smaller\;diameter\right)}^{2}$$

When tumors reached approximately 1.6 cm^3^ or at the end of the 21 day treatment period, the animals were euthanized, and tumors, organs, and blood samples were collected for histological and biochemical analyzes. Tumor and animal weights were monitored every 7 days using a precision scale.

### 2.4 Histological Analysis

Histological analysis was conducted to assess potential renal toxicity associated with MCP treatment. Kidneys from each group were collected after 21 days of treatment, sectioned, and immediately fixed in 4% buffered paraformaldehyde (pH 7.4) at 4 °C for 24 h. The samples were then dehydrated in a graded ethanol series (70–100%), with each step lasting 30 min, followed by three 30 min washes in xylene. The tissues were subsequently embedded in paraffin, sectioned into 4 μm slices, and stained with hematoxylin and eosin (H&E). Masson’s trichrome (MT) staining was also performed to visualize extracellular matrix deposits. Bright-field images were captured using LAS X Core Leica Microsystems Software (Leica Microsystems, Germany).

### 2.5 Biochemical Analysis

Biochemical analysis was conducted to assess potential hepatic and renal toxicity resulting from MCP treatment. Blood samples were collected from healthy animals and those treated with MCP or the vehicle (PBS) and centrifuged in a microcentrifuge (Hettich MIKRO 185) at 13,000 x *g* for 5 min to separate the plasma. The plasma samples were analyzed using the following reagent kits: Transaminase AST (TGO) (aspartate aminotransferase/oxalacetic transaminase) kinetic test (Bioclin KO48–9), Creatinine Automated kinetic test (Bioclin), Alkaline Phosphatase (ALP) IFCC kinetic test (Bioclin), Transaminase ALT (alanine aminotransferase) kinetic test (Bioclin KO49-6), Urea UV test (Bioclin K056-1), and Glucose Monoreagent Enzymatic Reagent (Bioclin KO82). All analyzes were performed using the ChemWell-T automatic biochemical analyzer (Labtest).

### 2.6 Radiolabeling of MCP and Quality Control

Radiolabeling of Modified Citrus Pectin (MCP) was performed as previously described, in a manner that preserves and mimics the native biological behavior of MCP in vivo.^43^ MCP (2.5 mg) was dissolved in saline (1 mL, 0.9% NaCl) and mixed with stannous chloride (20 μg; 4 mg/mL in 0.01 N HCl solution) under nitrogen gas for 5 min. Stannous chloride served as a reducing agent, converting ^99m^Tc from its oxidized state to a reactive form capable of binding to MCP. The pH was adjusted to 7 using 0.01 N NaOH, and sodium pertechnetate (Na­[^99^mTc]­O_4_^–^, 130 MBq) was added. The mixture was further purged with nitrogen for 5 min and incubated for 25 min. To ensure safety, all procedures were conducted in a hot laboratory under lead shielding. The concentrations of stannous chloride and pH were optimized to achieve high labeling efficiency (≥95%) while minimizing the formation of reduced/hydrolyzed ^99m^Tc (R/H ^99m^Tc), an undesirable byproduct.

- R (Reduced ^99m^Tc): Refers to ^99m^Tc reduced to a reactive form by stannous chloride, which may fail to bind MCP and instead form aggregates.
- H (Hydrolyzed ^99m^Tc): Forms when reduced ^99m^Tc reacts with water or other molecules, leading to colloidal particles that can nonspecifically accumulate in tissues.

The quality and purity of the radiolabeled MCP were assessed using ITLC-SG (Instant Thin-Layer Chromatography with Silica Gel; Agilent Technologies, CA, USA). Two mobile phases were used to distinguish the radiolabeled product ([^99m^Tc]­MCP) from byproducts: (1) 100% acetone; Free ^99m^TcO_4_^–^ migrates, while [^99m^Tc]­MCP and R/H ^99m^Tc remain at the origin; and (2) ethanol–ammonia–water (1:2:5): [^99m^Tc]­MCP and free ^99m^TcO_4_^–^ stay at the origin, while R/H ^99m^Tc migrates.

The percentages of free ^99m^TcO_4_^–^, R/H ^99m^Tc, and [^99m^Tc]­MCP were calculated, and radiochemical purity was determined using a PerkinElmer Wizard 2 2480 automatic γ-counter (Waltham, MA, USA). Preparations with radiochemical purity ≥ 95% were deemed suitable for experiments.

The labeling of PectaSol-C with ^99m^Tc is achieved through stannous chloride-mediated reduction of pertechnetate (^99m^TcO4−) to Tc­(IV/V)–oxo species, which can subsequently form coordinated bonds with donor groups within the polysaccharide. In modified citrus pectin, the most plausible coordination sites are the carboxylate groups of galacturonic acid residues, abundant in both homogalacturonan and rhamnogalacturonan domains, with additional stabilization from vicinal hydroxyl groups. This chelation mechanism accounts for the high radiochemical yields and the preferential labeling of the high-molecular-weight fraction.^31,37^

For IV studies, MCP (PectaSol-C) mass was kept low because bioavailability is 100%, whereas oral studies used a higher mass consistent with prior literature to offset low/variable absorption and first-pass metabolism. Injected radioactivity (^99m^Tc) was set to achieve comparable counting precision and imaging for each route: lower activity sufficed for IV; higher activity was required for oral to avoid subthreshold organ counts within the decay-limited acquisition window. Outcome measures were normalized to injected dose (e.g., %ID/g), and we report dose (MBq), injected MCP mass (mg/kg), and injected volume (μL) to document tracer levels (Table S3). All activities and masses remained within standard preclinical practice and were selected to avoid detector dead-time, radiolysis, or route-related formulation issues (viscosity/osmolality).

### 2.7 Stability Tests of [^99m^Tc]­MCP in Saline or Blood Plasma

Stability tests were conducted to evaluate the radiochemical stability of [^99m^Tc]­MCP. To quantify [^99m^Tc]­MCP, reduced/hydrolyzed ^99m^Tc (R/H ^99m^Tc), and free ^99m^TcO_4_^–^, stability tests were performed in saline and blood plasma. A solution of [^99m^Tc]­MCP (10 μL, 20 MBq) was added to 100 μL of saline (0.9% NaCl) or 100 μL of blood plasma obtained from heparinized (60 U/mL at 10%) female or male C57BL/6 mice. The plasma samples were prepared by centrifugation at 12,000*g* for 5 min to separate plasma from whole blood.

Samples were analyzed at multiple time points (0, 0.5, 1, 2, 3, 4, 5, 6, and 24 h) to evaluate the stability of the radiolabeled compound. Quality control was conducted using ITLC-SG (Instant Thin-Layer Chromatography with Silica Gel), as described in Section 2.5, to separate and quantify [^99m^Tc]­MCP, R/H ^99m^Tc, and free ^99m^TcO_4_^–^.

### 2.8 Stability of [^99m^Tc]­MCP at Different pH Levels

Stability tests at varying pH levels were conducted to assess the radiochemical stability of the molecule under conditions mimicking different physiological compartments, including low-pH environments such as the stomach. For pH stability assays, 5 mL of PBS buffer was prepared, and the pH was adjusted to 1, 2, 3, 4, 5, 6, 7, and 8 using 0.1 M HCl or 0.1 M NaOH. The pH levels were verified using a Kasvi pH meter (model K39-1420A).

The stability of [^99m^Tc]­MCP (20 MBq) at these varying pH levels was evaluated using ITLC-SG (Instant Thin-Layer Chromatography with Silica Gel), as outlined in Section 2.5. This analysis quantified the proportions of [^99m^Tc]­MCP, reduced/hydrolyzed ^99m^Tc (R/H ^99m^Tc), and free ^99m^TcO_4_^–^ to determine the compound’s stability across the tested pH range.

### 2.9 Stability of [^99m^Tc]­MCP In Vivo

Stability tests were conducted to evaluate the in vivo radiochemical stability of [^99m^Tc]­MCP, following the methodology described in a previous study.^43^ Male C57BL/6 mice were administered [^99m^Tc]­MCP (37 MBq) either intravenously or orally via gavage. Blood plasma samples were collected at specific time intervals: 0.01, 5, 15, 30, 45, and 60 min following intravenous administration, and 5, 30, 60, 120, and 240 min following oral administration.

Blood cells were separated from plasma by centrifugation at 12,000*g* for 5 min. To precipitate proteins in the plasma supernatants, methanol (100 μL) was added to each sample, followed by centrifugation at 12,000*g* for another 5 min.

In vivo stability design and sampling windows. After IV administration, the early distribution phase (0–60 min) was sampled because any ^99m^Tc dissociation would be most apparent during first-pass circulation; later time points yield lower plasma activity (distribution + physical decay) under mouse blood-volume limits, reducing precision without improving the study end points. After oral dosing, sampling was extended to 4 h to cover gastrointestinal transit and delayed systemic appearance. In vitro stability was assessed in mouse plasma to 24 h to complement the in vivo windows. All blood sampling volumes complied with institutional guidelines and 3Rs principles.

The stability of the [^99m^Tc]­MCP complex in plasma (5 μL) was assessed using ITLC-SG (Instant Thin-Layer Chromatography with Silica Gel) as described in Section 2.5, enabling quantification of [^99m^Tc]­MCP, reduced/hydrolyzed ^99m^Tc (R/H ^99m^Tc), and free ^99m^TcO_4_^–^.

### 2.10 Kinetic Studies

Kinetic studies were conducted to assess the in vivo absorption of MCP. These studies were performed using [^99m^Tc]­MCP in male C57BL/6 mice. For the intravenous study, 100 μL of [^99m^Tc]­MCP (10–20 MBq, ∼0.3 mg) was filtered through a Millex-GV Millipore 0.22 μm filter and injected into the tail vein. For the oral study, 37 MBq of [^99m^Tc]­MCP (∼0.7 mg) was administered by oral gavage.

Venous blood samples were collected at designated time intervals: 0.01, 5, 10, 15, 30, 60, 120, 240, and 1440 min postinjection for the IV study, and 5, 30, 60, 120, and 240 min for the oral study. The activity in blood samples was measured using a PerkinElmer Wizard 2 2480 automatic γ-counter (Waltham, MA, USA), with radiological activity corrected for the radionuclide half-life.

### 2.11 Bioavailability Studies

Bioavailability was used to quantify the in vivo absorption of MCP. Bioavailability (F) was calculated using the plasma concentration–time curve,^44^ which includes the assessment of the maximum plasma concentration (*C*_max_), the time to reach peak concentration (*T*_max_), and the area under the curve from time zero to infinity (AUC_0–∞_). Bioavailability following oral administration was determined using the following formula

2 $$F={[Dose}_{i.v.}\times {AUC}_{i.g.}/{Dose}_{i.g.}\times {AUC}_{i.v.}]\times 100\%$$

where Dose_i.g._: the oral administration dose; Dose_i.v._: the intravenous dose; AUC_i.g._: the AUC_0–∞_ after oral administration; AUC_i.v._: the AUC_0–∞_ after intravenous injection.

### 2.12 Biodistribution Studies

Biodistribution studies were conducted to determine the in vivo distribution profile of MCP at specific time points. *Lgals3* ^–/–^ C57BL/6 mice were generated as previously described^45^ and obtained from the breeding colony at the Federal University of Rio de Janeiro, Brazil. All experimental procedures were conducted in accordance with the ARRIVE guidelines and the Guidance on the Operation of the Animals (Scientific Procedures) Act 1986 and were approved by the Institutional Animal Ethics Committee (Protocol numbers DAHEICB-071, 16/22, and 2021/05). In biodistribution studies, male C57BL/6*Lgals3* ^+/+^ and C57BL/6*Lgals3* ^–/–^ mice, as well as BALB/c nude mice with xenografts, were administered [^99m^Tc]­MCP either intravenously (10 MBq) via tail vein injection or orally (37 MBq) via gavage.

One hour after administration, the mice were euthanized, and organs of interest were harvested. The organs were rinsed in PBS, weighed, and analyzed for radioactivity using a PerkinElmer Wizard 2 2480 automatic γ-counter (Waltham, MA, USA). The data were expressed as the percentage of the injected dose per gram of tissue (% ID/g), providing insights into the distribution of [^99m^Tc]­MCP across various tissues.

### 2.13 μSPECT/CT Imaging

μSPECT/CT imaging was performed to visualize the in vivo localization of MCP at specific time points. For imaging, [^99m^Tc]­MCP (37 MBq) was administered either intravenously or orally (via gavage) following 6 h fasting period in male and female BALB/c nude mice with xenografts. Imaging was conducted at 1 and 4 h postinjection using an Albira μSPECT/CT system (Bruker Biospin Corporation, Woodbridge, CT, USA). The acquired images were processed and quantified using PMOD software (PMOD Technologies, Zurich, Switzerland).

### 2.14 Autoradiography

Autoradiography was performed to determine the intratumoral localization of MCP within the xenograft at specific time points. Tumors harvested (SKOV-3-derived tumors) from male and female BALB/c nude mice were frozen, sectioned using a cryostat, and mounted on Superfrost Plus microscope slides. The tumor sections were exposed to a phosphor screen, incubated overnight, and subsequently scanned using a Typhoon FLA 9500 scanner (GE Healthcare, Chicago, IL, USA). Necrotic regions within the tumor sections were quantified using ImageJ software.

### 2.15 Production of MCP Fractions

Production of MCP fractions was carried out to investigate the molecular properties of MCP. Fractionation was performed as previously described in literature.^46^ Briefly, MCP samples (prepared in triplicate) were water-solubilized and fractionated into different molecular size ranges through sequential ultrafiltration using 30, 10, and 3 kDa MWCO Amicon Ultra-4 Centrifugal Filters (Millipore). The resulting filtrates and retentates were lyophilized, yielding four distinct MCP fractions: (1) MCP > 30 kDa, (2) MCP between 30 and 10 kDa (MCP < 30 > 10 kDa), (3) MCP between 10 and 3 kDa (MCP < 10 > 3 kDa), and (4) MCP < 3 kDa.

### 2.16 Molecular Weight and Homogeneity

Molecular weight and homogeneity analyzes were conducted to determine the size distribution and structural uniformity of MCP. Samples from each MCP fraction (3 mg) were dissolved in 1 mL of deionized water, and molecular weight distribution was analyzed using high-performance size-exclusion chromatography with refractive index detection (HPSEC-RID). The analysis was performed on an Infinity 1250 system (Agilent, Santa Clara, CA, USA) equipped with four PL aquagel–OH columns (60, 50, 40, and 30; 300 × 7.5 mm; Agilent) connected in tandem. The eluent was 0.2 M NaNO_3_ containing 0.02% NaN_3_, delivered at a flow rate of 0.6 mL/min, with the RID detector temperature set at 30 °C.

Molecular sizes were estimated using the Dextran T-series standards (25, 50, 80, 150, 410, and 750 kDa; Sigma, St. Louis, MO, USA) as references, allowing for precise determination of molecular weight ranges in the MCP fractions.^44^

### 2.17 Monosaccharide Hydrolysis and Polysaccharide Composition

Monosaccharide hydrolysis and compositional analysis were performed to determine the monosaccharide profile of MCP fractions. Each fraction (1 mg) was hydrolyzed using 2 M trifluoroacetic acid (TFA) to release constituent monosaccharides. Following hydrolysis, the samples were dried, resuspended in water, and analyzed for their neutral sugar and uronic acid composition using high-performance anion-exchange chromatography with pulsed amperometric detection (HPAEC-PAD). The analysis was performed on an ICS5000+ system (Thermo-Dionex, Waltham, MA, USA). Neutral sugars, including l-arabinose, d-galactose, d-glucose, d-fucose, d-mannose, l-rhamnose, and d-xylose, as well as uronic acids (d-glucuronic and D-galacturonic acids), were used as external standards (Sigma, St. Louis, MO, USA), as described in.^46^

MCP (PectaSol-C) was fractionated by ultrafiltration into (1) MCP > 30 kDa, (2) MCP between 30 and 10 kDa (MCP < 30 > 10 kDa), (3) MCP between 10 and 3 kDa (MCP < 10 > 3 kDa), and (4) MCP < 3 kDa pools. Each pool was analyzed for molecular weight distribution and monosaccharide composition (method 2.16, 2.17), but glyco-epitope mapping (e.g., RG-I vs HG content) and metabolite identification after oral dosing were not performed in this study.

### 2.18 SDS-PAGE Gel System

SDS-PAGE was performed to assess the structural integrity of Gal-3. Proteins (50 μg) were separated using the Novex NuPAGE SDS-PAGE gel system (Invitrogen) with a 12% acrylamide gel. A Full Ranger Rainbow molecular weight ladder (Z892 53410) was used as a standard, and protein bands were visualized with Bio-Safe Coomassie blue stain (1610786). Band analysis was conducted using ImageJ software for quantification and visualization.

### 2.19 Hemagglutination Assay

A hemagglutination assay was conducted to evaluate the affinity and interaction between MCP and Gal-3. Recombinant Gal-3 was produced as described in literature,^47^ and the assay was performed according to the protocols outlined in.^48,49^ Briefly, erythrocytes were collected via cardiac puncture from male and female C57BL/6 mice, isolated, and prepared as a 3% suspension. Each well of a V-plate contained 100 μL of Gal-3 (1–20 μM), 3% erythrocytes, 1% bovine serum albumin (BSA), and the sample diluted in PBS containing sucrose (10–100 mM), lactose (12.5, 25, 50, and 100 mM), or MCP fractions (MCP, MCP < 3 kDa, MCP > 30 kDa, MCP < 30 > 10 kDa, or MCP < 10 > 3 kDa) at concentrations of 5–25 mg/mL. The plates were incubated at room temperature for 120 min, and hemagglutination was assessed by observing button formations at the center of the wells.

### 2.20 Sepharose/Gal3 Column Assay

An affinity chromatography assay was performed using a Sepharose/Gal-3 column to assess the binding interaction between MCP and Gal-3. The column was prepared by coupling recombinant Gal-3 to cyanogen bromide-activated Sepharose (CNBr-activated Sepharose, C9142-5G). The column was first washed with 20 mL of PBS to remove unbound material. [^99m^Tc]­MCP or [^99m^Tc]­MCP > 3 kDa (1 mg/mL containing 37 MBq) was then added to the column, and the system was incubated for 5 min with both ends capped to allow binding. Following incubation, the column was washed with 15 mL of PBS under continuous flow for 15 min to remove nonspecifically bound material. Subsequently, the column was washed with 15 fractions of 100 mM lactose (1 mL each) under constant flow to elute specifically bound [^99m^Tc]­MCP or [^99m^Tc]­MCP < 3 kDa. All PBS and lactose fractions were collected in 1.5 mL Eppendorf tubes, and the radioactivity of each fraction was measured using a PerkinElmer Wizard 2 2480 automatic γ-counter (Waltham, MA, USA). Radioactivity measurements were corrected for the radionuclide half-life, and the percentage of elution with lactose was calculated to evaluate specific binding and elution efficiency.

### 2.21 Pharmacokinetic Studies

Pharmacokinetic studies were conducted to evaluate the in vivo pharmacokinetic profile of MCP. These studies were performed in male C57BL/6*Lgals3* ^+/+^ and C57BL/6*Lgals3* ^–/–^ mice. [^99m^Tc]­MCP (10–20 MBq in 100 μL) was administered via tail vein injection, and venous blood samples (5 μL) were collected from the tail vein at specified time points: 0.01, 5, 10, 15, 30, 60-, 120-, 240-, and 1440 min postinjection (p.i.). The radioactivity in each blood sample was measured using a PerkinElmer Wizard 2 2480 automatic γ-counter (Waltham, MA, USA). Radioactivity data were corrected for the radionuclide half-life, and correlations were made with the injected activity and mass. The distribution and elimination half-lives were determined using nonlinear exponential regression based on the “two-phase decay” model and a two-dimensional exponential plot, which allowed for the calculation of both the distribution and elimination half-lives. Clearance (CL) was calculated using the following formula

CL=totalinjectedmass(mg)/AUC

where: AUC = area under the plasma concentration–time curve, determined using statistical x/y analysis.

The volume of distribution (*V*_d_) was calculated using the equation

$$V_{d}=CL\times T_{1/2}/0.693$$

Where: *T*_1/2_ is the elimination half-life.

These calculations provided insight into the pharmacokinetics and bioavailability of [^99m^Tc]­MCP in the studied mouse models.

### 2.22 Blood Compartment Distribution Assay

A blood compartment distribution assay was conducted to assess the binding of MCP to blood cells, plasma proteins, and other components in vivo. The assay was performed according to the methodology described in literature.^50^ Male C57BL/6^+/+^ and C57BL/6*Lgals3* ^–/–^ mice were intravenously injected with 15 MBq of [^99m^Tc]­MCP. Venous blood samples (10 μL) were collected from the tail vein at 5, 15, 30, 45, and 60 min postinjection (p.i.) and processed as outlined in Section 2.19. The samples were fractionated to separate blood cells, proteins, and plasma, and the radioactivity in each fraction was measured using a PerkinElmer Wizard 2 2480 automatic γ-counter (Waltham, MA, USA). All radioactivity measurements were corrected for the radionuclide half-life to ensure accuracy.

### 2.23 Partition Coefficient

A partition coefficient assay was performed to assess the solubility and lipophilicity of MCP. The partition coefficient of [^99m^Tc]-MCP was determined using a two-phase system consisting of physiological saline (0.9% NaCl) as the aqueous phase and *n*-octanol as the organic phase, representing hydrophilic and lipophilic environments, respectively.^51^ Briefly, an Eppendorf tube containing 450 μL of 0.9% NaCl, 50 μL of [^99m^Tc]­MCP (1 MBq), and 500 μL of *n*-octanol was mixed. The tube was capped and shaken vigorously for 5 min at room temperature. Following mixing, the solution was centrifuged at 2000*g* for 2 min to separate the phases. Aliquots (100 μL) from each phase were collected, and the radioactivity in the aqueous (saline) and organic (*n*-octanol) phases was measured using a PerkinElmer Wizard 2 2480 automatic γ-counter (Waltham, MA, USA). The experiments were conducted in triplicate, and the partition coefficient (*P*) was calculated using the following equation

P=c_org/c_aq

Where: c_org: is the radioactivity measured in the *n*-octanol (organic phase), c_aq: is the radioactivity measured in the saline solution (0.9% NaCl, aqueous phase).

The logarithm of the partition coefficient (log *P*) was then calculated to indicate the compound’s relative hydrophilicity or lipophilicity.

### 2.24 Immunostaining Assay

Immunostaining was performed as described in literature.^52^ Immunohistochemistry (Gal-3). Tumors harvested after biodistribution were embedded and cryosectioned (8–10 μm). Sections were fixed (e.g., acetone or 4% PFA), blocked, and incubated with rat anti- Gal-3 primary antibody (clone M3/38) followed by biotinylated antirat IgG secondary (Vector BA-4001) and streptavidin-peroxidase (Sigma). Signal was developed with DAB (DAKO) and counterstained with hematoxylin/eosin. Images were acquired on TissueFAXS and analyzed in ImageJ/QuPath. For each tumor, ≥3 nonoverlapping fields were quantified; Gal-3-positive area fraction or H-score was recorded. Negative controls (isotype/no primary) were included.

### 2.25 Statistical Analysis

All data are presented as the mean ± standard deviation (SD) from at least three independent experiments. Statistical analysis was conducted using GraphPad Prism software (version 8.0; San Diego, CA, USA). Unpaired *t* tests (multiple *t* tests) were used for comparisons, with outliers identified and removed prior to analysis. Statistical significance was defined as *p* < 0.05.

## 3 Results and Discussion

### 3.1 Intravenous MCP Reduces Tumor Growth in a Xenograft Model, while Oral Administration Lacks Efficacy

To evaluate the antitumor effects of intravenously and orally administered MCP, we established SKOV-3 and MKN45 tumor xenografts and 4T1 syngeneic (BALB/c) tumor grafts in mice. The MCP doses used for IV and oral treatment were selected based on prior in vivo studies.^14,22,44–50^ When tumors reached ∼50 mm^3^, mice were treated daily with MCP (IV: 10 mg/kg; oral: 200 mg/kg) for 21 days, or for 15 days in the 4T1 studies due to the faster growth rate of 4T1 tumors. Notably, IV-administered MCP (10 mg/kg) reduced tumor growth by approximately 48.5% and tumor weight by 50% in SKOV-3-bearing mice, without a significant impact on body weight (Figure 1A–C). Similar reductions were observed in the MKN45 xenograft model and the 4T1 syngeneic model (Figure S1A–F). In contrast, oral MCP (200 mg/kg) did not significantly alter tumor growth or weight in the SKOV-3 xenograft model (Figure 1D–F) or in the 4T1 syngeneic model (Figure S1) and was associated with biochemical evidence of liver and kidney injury (Figure S1 J,K; Table 1). Collectively, these findings indicate that IV MCP at 10 mg/kg decreases tumor burden across xenograft and syngeneic models under the conditions tested, consistent with an antitumor effect that does not require a predominant contribution from adaptive immunity, whereas oral MCP not only fails to reduce tumor burden but may also induce organ toxicity. These findings pertain to SKOV-3, MKN45, and 4T1 under the doses, timing, and conditions tested. The SKOV-3 xenograft model was selected for the bioavailability and biodistribution studies.

**Figure 1:**
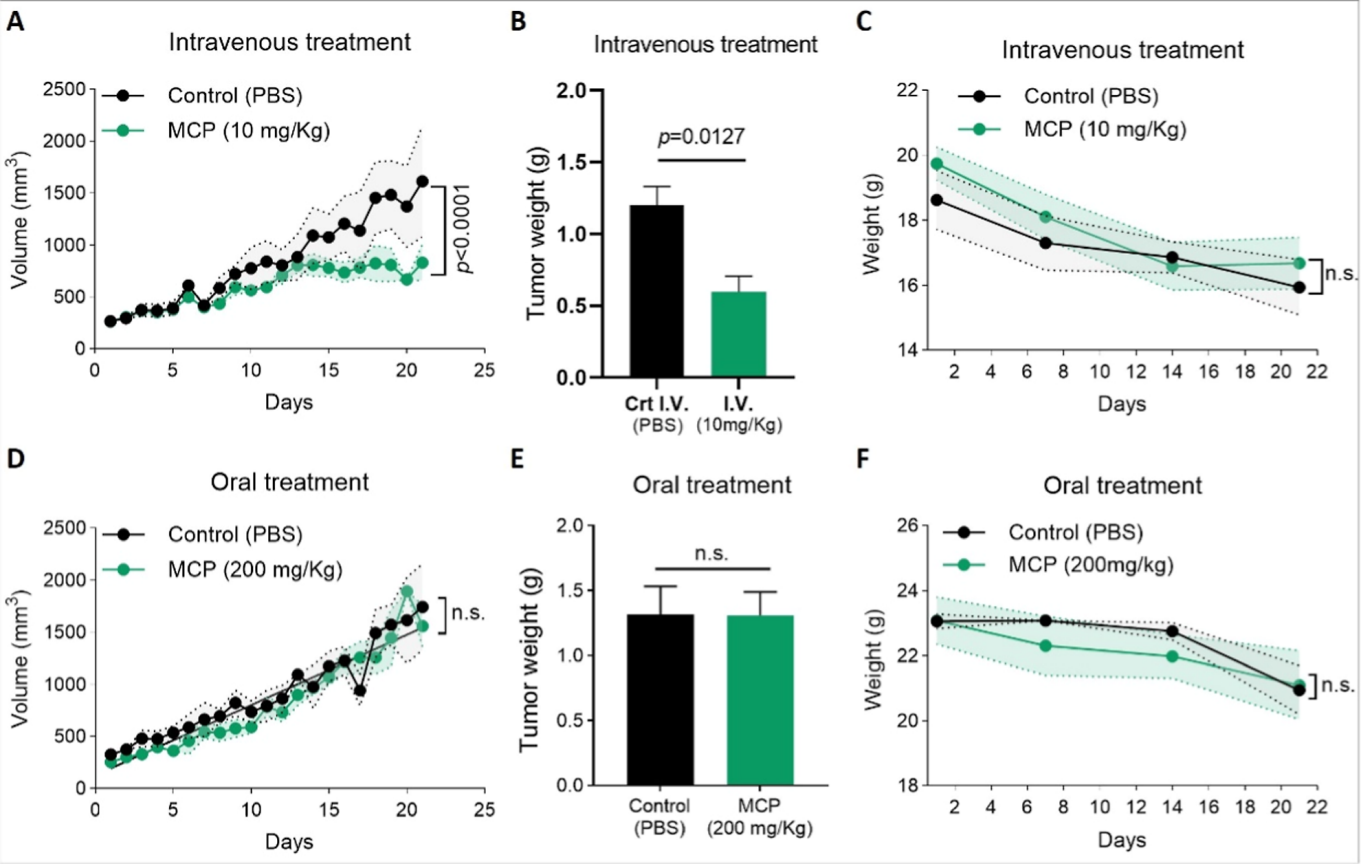
Intravenous, but not oral, administration of MCP (PectaSol-C) inhibits tumor growth in SKOV-3 xenograft models. (A) Tumor volume progression following intravenous treatment with vehicle (Control, PBS) or MCP (10 mg/kg) daily for 21 days in BALB/c nude mice bearing subcutaneous SKOV-3 tumors. Tumor volume (mm^3^) was measured daily (B) Excised tumor weight (g) at the end of the study period for the IV treatment groups. (C) Animal body weight (g) monitoring of animals bearing SKOV-3 tumor xenografts following intravenous MCP administration. (D) Tumor volume progression following oral gavage treatment with vehicle (Control, PBS) or MCP (200 mg/kg). (E) Excised tumor weight (g) at the end of the study period for the oral treatment groups. (F) Animal body weight (g) monitored throughout the oral treatment period. MCP = PectaSol-C (modified citrus pectin). Data are presented as the mean ± SD of *n* = 5.

**Table 1:** Bioavailability of [^99m^Tc]­MCP^a^

Administration	AUC (% ID)	*C*_max_ (% ID)	*T*_max_ (min)	*F* (%)
Intravenous	7.1 ± 2.1	1.3 ± 0.2	0.1	-
Oral	9.4 × 10^–6^ ± 9.1 × 10^–7^	5.3 × 10^–6^ ± 7.5 × 10^–7^	60	4.3 × 10^–5^

a AUC_0–∞_, the area under the curve from time zero to infinity; Cmax, peak plasma concentration; *T*_max_, the time to reach the peak concentration; *F*, bioavailability.

MCP is most commonly studied via oral administration in preclinical and limited clinical settings, but other routes (intravenous, intraperitoneal, intratumoral, intrathecal, and intracerebroventricular) have also been investigated. Previous studies have reported oral MCP benefits in models of melanoma, breast carcinoma, colon carcinoma,^22,23^ bladder tumor,^19^ colorectal cancer,^24^ prostate cancer,^25^ breast cancer,^14^ and thyroid carcinoma.^26^ However, a 20% MCP diet over 21 days yielded negative outcomes in a colorectal cancer model.^27^ In alignment with our present findings, intravenous or alternative MCP administration routes have shown anticancer activity in metastatic breast and prostate cancer,^38^ melanoma,^39^ mammary adenocarcinoma metastasis,^53^ and colon cancer.^40^

It is important to note that, in contrast to subcutaneous graft models, orally administered MCP in chemically induced tumor models (e.g., azoxymethane- or methylnitrosourea-treated rodents) may yield different outcomes.^54–56^ Some studies have demonstrated that dietary pectins or MCP can reduce cancer incidence and tumor size in colon cancer models.^57^ Conversely, one report noted that citrus pectin and MCP did not protect against azoxymethane–dextran sodium sulfate–induced tumors.^27^ Collectively, these data indicate that MCP’s efficacy may depend on factors such as tumor model, induction method, and administration route.

### 3.2 [^99m^Tc]­MCP Exhibits Low Absorption when Administered Orally

To investigate MCP’s absorption, distribution, and pharmacokinetics, we radiolabeled MCP with ^99m^Tc, achieving >95% radiochemical purity and stability for up to 5 h in saline and 24 h in plasma (Figures 2A,B; Figure S2A–D). In vivo stability remained >95% at 1 h post-IV injection and >87% at 4 h postoral injection, indicating partial degradation in the gastrointestinal tract (Figure 2C,D).

**Figure 2:**
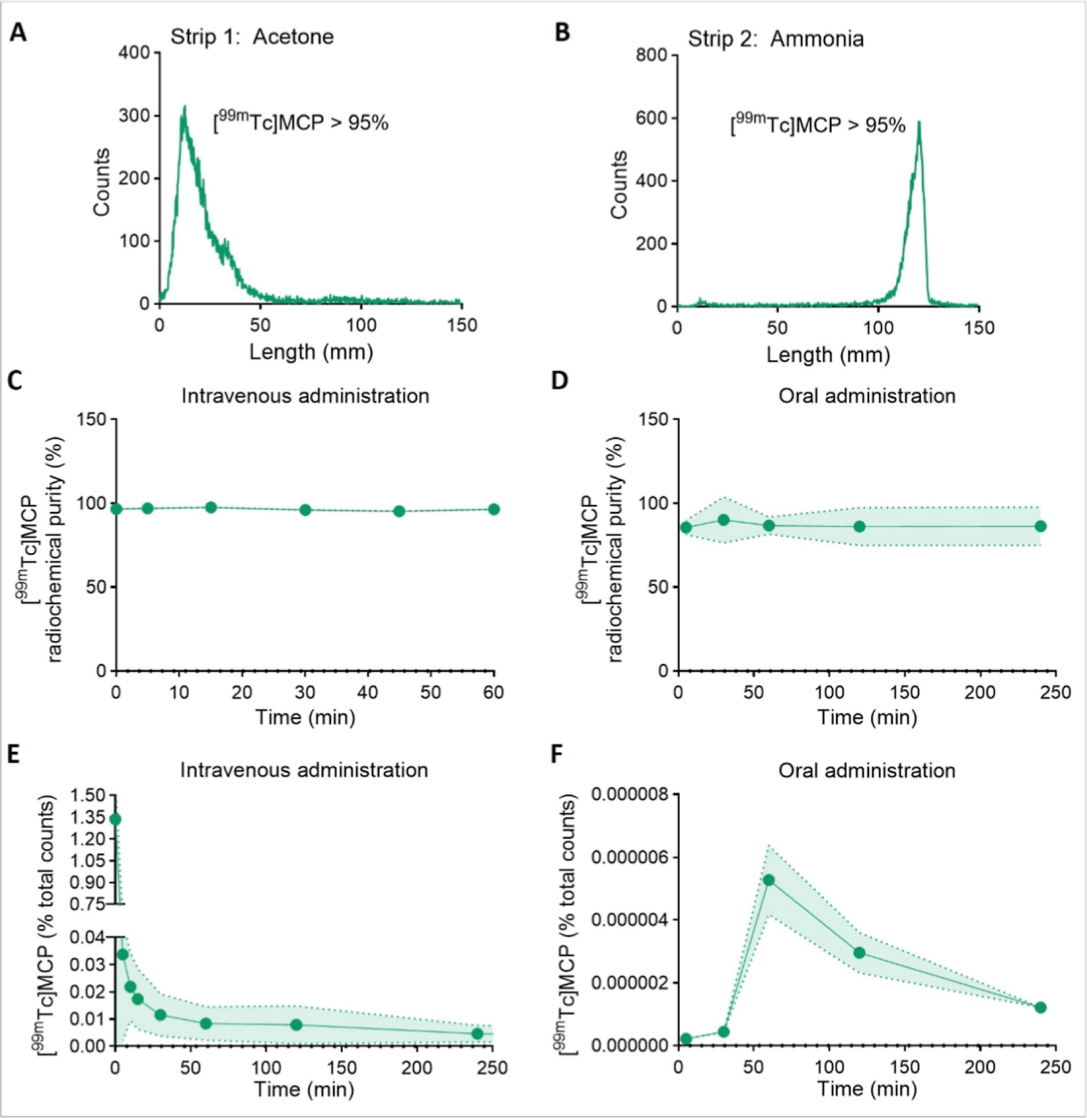
[^99m^Tc]­MCP exhibits low absorption when administered orally. (A,B Quality control of [^99^ ^m^Tc]­MCP radiolabeling using Instant Thin-Layer Chromatography (ITLC-SG). Representative chromatograms show radiochemical purity >95%. (C) In vivo stability of [^99^ ^m^Tc]­MCP in mouse plasma over 60 min following intravenous administration, assessed by ITLC-SG. (D) In vivo stability of [^99^ ^m^Tc]­MCP in mouse plasma over 240 min following oral administration, assessed by ITLC-SG. (E) Pharmacokinetic profile of [^99^ ^m^Tc]­MCP in blood following IV administration (15 MBq) in male C57BL/6 mice, showing percentage of injected dose (%ID) over time. (F) Pharmacokinetic profile of [^99^ ^m^Tc]­MCP in blood following oral administration (37 MBq) in male C57BL/6 mice, showing extremely low absorption. MCP = PectaSol-C (modified citrus pectin). Data are presented as mean ± SD, *n* = 5 animals per group.

Comparative kinetic studies revealed stark differences between IV and oral MCP absorption. After IV injection, [^99m^Tc]­MCP reached a *C*_max_ of 1.3 ± 0.24% ID at 0.1 min (*T*_max_) and had an elimination half-life of 502.89 ± 35.37 min (Figure 2E, Table 2). In contrast, oral administration yielded minimal absorption (*C*_max_ of 5.27 × 10^–6^ ± 7.5 × 10^–7^% ID) with a *T*_max_ of 60 min and extremely low bioavailability (4.3 × 10^–5^%) (Figure 2F, Table 1). Because GI depolymerization may occur, oral measurements represent [^99m^Tc]­MCP-derived species detected systemically, not necessarily intact MCP. These data confirm poor gastrointestinal uptake of MCP, limiting its efficacy via the oral route.

**Table 2:** [^99m^Tc]­MCP Pharmacokinetic Parameters in C57BL/6 *Lgals3* ^+/*+*^ and *Lgals3* ^–/*–*^ ^a^

PK parameter	*Lgals3* ^+/+^	*Lgals3* ^–/–^	*p* value
*T*_1/2α_ (min)	0.77 ± 0.06	2.40 ± 1.06	0.1653
*T*_1/2β_ (min)	502.89 ± 35.37	303.53 ± 30.63	0.0027
CL (μL/min)	1693.64 ± 368.39	2863.14 ± 129.40	0.0172
*V*_d_ (μL)	1,007,865 ± 496,145	1,023,093 ± 465,654	0.9827

a Parameters: distribution half-life (*T*_1/2α_), elimination half-life (*T*_1/2β_), clearance (CL), and volume of distribution (*V*_d_) of [^99m^Tc]­MCP in *Lgals3* ^+/+^ and *Lgals3* ^–/–^ mice. Data are representative of three independent experiments.

To our knowledge, technetium-99m radiolabeling of MCP (PectaSol-C) used to study in vivo biodistribution has not been previously described. Previous studies have used similar labeling strategies for other polysaccharides, such as Mannan-^99m^Tc, which showed comparable radiochemical purity.^43^ Additionally, pectin labeled with ^99m^Tc has been explored in colon scintigraphy, underscoring the utility of radiolabeled pectins for intestinal flow studies.^58,59^

Proposed mechanisms for orally administered MCP include modulating gut immune barriers, influencing intestinal microbiota, or indirectly interacting with immune cells.^60,61^ While MCP can be degraded by colonic enzymes into short-chain fatty acids that can impact the immune system,^62^ systemic absorption of intact MCP appears limited. Nevertheless, some studies suggest low-level absorption or induction of circulating antibodies against MCP fragments.^63,64^ Our results did not show tumor reduction under oral administration in a xenograft model, but additional research is needed to clarify MCP’s oral efficacy under different conditions.

### 3.3 [^99m^Tc]­MCP is Cleared via Renal and Hepatobiliary Pathways and Accumulates in Tumors at Low Levels

To assess MCP’s capacity to reach tumor tissue, we performed biodistribution and μSPECT imaging in SKOV-3 tumor-bearing mice. Animals received [^99m^Tc]­MCP either IV (10 MBq) or orally (37 MBq). One hour postadministration, organs were collected for analysis of the percentage of injected dose per gram (% ID/g) (Figure 3A).

**Figure 3:**
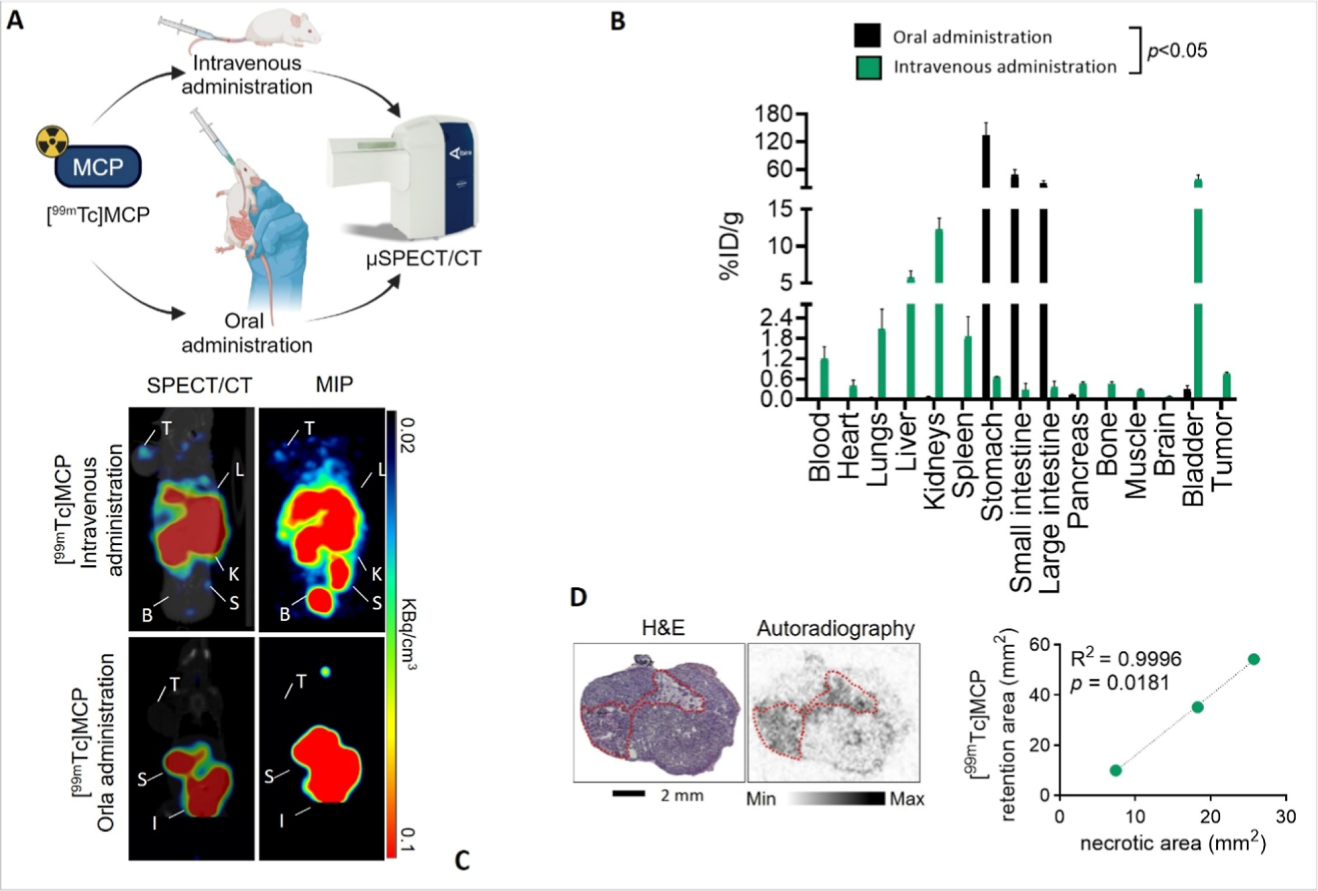
Biodistribution and tumor localization of [^99^ ^m^Tc]­MCP show renal/hepatobiliary clearance and low tumor uptake, particularly after oral administration. (A) Schematic illustrating the experimental setup for in vivo μSPECT/CT imaging and biodistribution analysis following either intravenous or oral administration of [^99^ ^m^Tc]­MCP. (B) Biodistribution analysis of [^99m^Tc]­MCP 1 h post oral or intravenous administration. Data show the percentage of injected dose per gram of tissue (%ID/g) in selected organs. (C) Representative μSPECT/CT imaging of [^99m^Tc]­MCP acquired 1 h post oral or intravenous administration. *T* = tumor, L = liver, K = kidney, B = bladder, S = spleen, I = intestine, MIP = maximum intensity projection. (D) Left: Hematoxylin and Eosin (H&E) staining of a representative SKOV-3 tumor section showing viable and necrotic areas. Right: Corresponding autoradiography image showing [^99^ ^m^Tc]­MCP localization (higher intensity signal) predominantly within necrotic regions (indicated by dotted lines). MCP = PectaSol-C (modified citrus pectin). Graph shows quantification correlating [^99^ ^m^Tc]­MCP retention area with necrotic area (mm^2^), with R^2^ = 0.9996 and *p* = 0.0181. Data in B and D graph are mean ± SD, *n* = 5 animals. Images in C and D are representative of *n* = 3 independent experiments.

IV-administered [^99m^Tc]­MCP reached tumor tissue at low concentrations (0.78 ± 0.045% ID/g), while oral administration yielded minimal tumor uptake (0.015 ± 0.005%ID/g). IV injection led primarily to renal and hepatobiliary excretion (with significant uptake in kidneys, bladder, and liver), whereas oral administration predominantly followed the gastrointestinal route (high stomach and intestinal activity) (Figure 3B). A similar result was also observed in the MKN45 tumor xenograft models (Figure 2E). μSPECT/CT imaging confirmed these elimination pathways and demonstrated slightly higher tumor uptake with IV administration (Figure 3C, Figure 3, Table 2). Autoradiography localized IV [^99m^Tc]­MCP mainly to areas of cell death within tumors (Figure 3D).

Our findings demonstrate that oral administration of MCP results in negligible systemic absorption and minimal tumor uptake, as evidenced by its extremely low bioavailability (<0.01%) and lack of antitumor efficacy in the xenograft model. This contrasts sharply with intravenous administration, which achieved measurable antitumor effects despite low levels of MCP reaching the tumor microenvironment.

These data concur with earlier work on Mannan-^99m^Tc, which exhibited similar excretion patterns.^43,65^ Notably, MCP can be metabolized by certain gut bacteria (e.g., *Monoglobus pectinilyticus*), although such microbial activity does not appear to enhance systemic absorption.^66–68^ Overall, our data suggest that although IV-administered MCP can reach tumors at low levels, oral MCP has negligible tumor uptake.

Interestingly, the small amounts of MCP that localized to tumors were primarily confined to necrotic regions, suggesting that MCP’s antitumor effects are unlikely to be mediated by direct or indirect actions within the tumor microenvironment itself. These results raise important considerations for ongoing clinical trials that utilize MCP or related pectin formulations administered orally.

Specifically, our data highlight that the therapeutic potential of MCP may not rely on direct interactions with tumor cells or their microenvironment. Instead, its systemic antitumor effects may involve alternative mechanisms occurring outside of the tumor site. While oral MCP may offer benefits related to gut health or localized gastrointestinal conditions, our study suggests that systemic antitumor efficacy requires intravenous administration to bypass absorption barriers. Importantly, the observed antitumor effects in immune-deficient animals further suggest that MCP’s efficacy may not depend on immunomodulation, as the animals lacked a fully functional immune system. Given these findings, ongoing and future clinical trials should carefully consider the following adjustments: (1) Dosing Strategies: Higher oral doses may not compensate for the fundamental limitations in absorption and systemic delivery. Alternative strategies, such as encapsulation in nanoparticles or coadministration with absorption enhancers, may be required to improve bioavailability; (2) Route of Administration: Exploration of nonoral routes, such as intravenous or intraperitoneal delivery, could maximize MCP’s therapeutic potential by bypassing the gastrointestinal barrier; (3) Target Indications: Oral MCP may be better suited for localized gastrointestinal conditions or diseases where systemic distribution is not critical. Trials focused on colorectal or intestinal cancers, where direct interaction with the gut epithelium or modulation of the gut environment is beneficial, could be prioritized and; (4) Mechanistic Studies: MCP may influence systemic factors such as hormone levels, cytokines, or circulating growth factors, indirectly creating an environment less conducive to tumor growth.

Given these findings, ongoing and future clinical trials should carefully consider these potential mechanisms and prioritize studies that explore nonoral routes of administration, such as intravenous or intraperitoneal delivery, to maximize MCP’s therapeutic potential. This nuanced understanding of MCP’s mechanisms, particularly its ability to exert antitumor effects independently of immune system modulation, offers valuable insights for designing future trials and identifying complementary therapeutic strategies.

### 3.4 MCP Comprises Diverse Monosaccharides and Shows Limited Galectin-3 Binding In Vitro

To explore the structural makeup of MCP and its affinity for Gal-3, we fractionated MCP by molecular weight into four fractions: MCP > 30 kDa, MCP < 30 > 10 kDa, MCP < 10 > 3 kDa, and MCP < 3 kDa. Analysis HPAEC-PAD revealed that most fractions consisted primarily of galacturonic acid (GalA), with varying amounts of arabinose (Ara), rhamnose (Rha), and galactose (Gal), particularly in MCP > 30 kDa and MCP < 3 kDa (Figure 4A, Supplementary Figure 4A).

**Figure 4:**
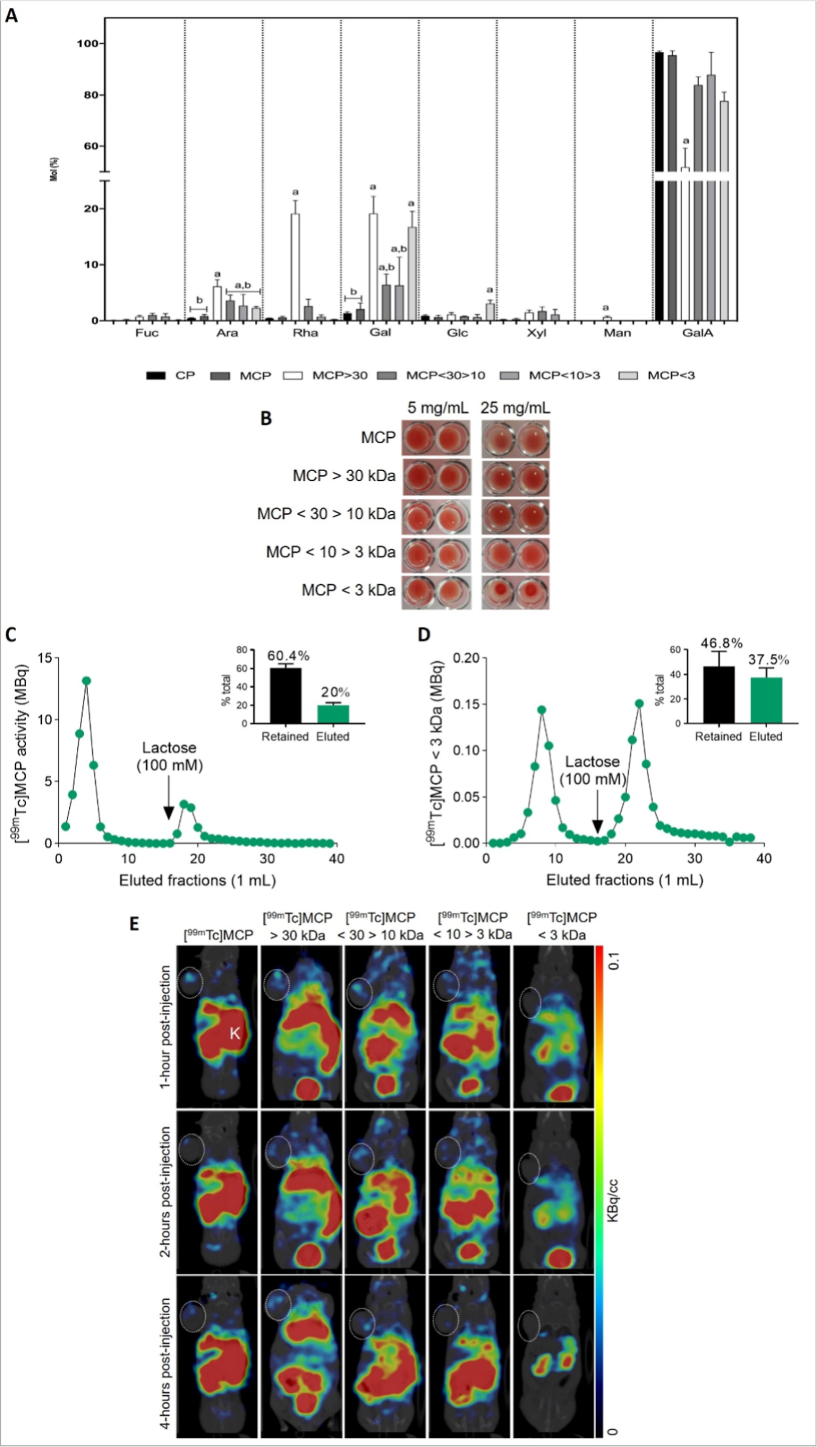
MCP fractions exhibit diverse monosaccharide composition and demonstrate low affinity for galectin-3. (A) Monosaccharide composition (mol %) of unfractionated MCP and its fractions obtained by ultrafiltration (MCP > 30 kDa, MCP < 30 > 10 kDa, MCP < 10 > 3 kDa, and MCP < 3 kDa) analyzed by HPAEC-PAD. Bars represent l-arabinose (Ara), d-galactose (Gal), d-glucose (Glc), d-fucose (Fuc), d-mannose (Man), l-rhamnose (Rha), d-xylose (Xyl), along with uronic acids d-glucuronic acid and d-galacturonic acid (GalA). Statistical differences (*p* < 0.05) between fractions for specific monosaccharides are indicated by letters (a, b, c). (B) Hemagglutination inhibition assay showing interaction between recombinant Gal-3 (fixed concentration) and varying concentrations (5 mg/mL vs 25 mg/mL) of MCP or its fractions. (C) Gal-3 affinity chromatography using [^99^ ^m^Tc]­MCP in Sepharose/Gal-3 column. (D) Gal-3 affinity chromatography using [^99^ ^m^Tc]­MCP < 3 kDa in Sepharose/Gal-3 column. (E) Representative SPECT/CT imaging 1 h post intravenous injection of [^99m^Tc]­MCP or its radiolabeled fractions ([^99m^Tc]­MCP > 30, [^99m^Tc]­MCP < 30 > 10, [^99m^Tc]­MCP < 10 > 3, and [^99m^Tc]­MCP < 3 in BALB/c nude mice bearing SKOV-3 tumors. Images show kidneys (K) and tumor region (indicated by crosshatched circles). MCP = PectaSol-C (modified citrus pectin). Data in A, C, D are mean ± SD, *n* = 3 independent experiments. Images in B and E are representative of *n* = 3 experiments.

When tested for Gal-3 binding, MCP < 3 kDa inhibited Gal-3 at high concentrations (25 mg/mL) and showed marginally greater affinity than other fractions. Approximately 60% of total [^99m^Tc]­MCP retained on a Gal-3 column was eluted with lactose (20%), whereas MCP < 3 kDa displayed slightly higher lactose-eluted activity (∼37.5%), indicating enhanced Gal-3 binding (Figure 4B–D, Figure S4B–D).

Despite these in vitro indications, SPECT/CT imaging did not reveal noticeable tumor accumulation of MCP < 3 kDa or other MCP fractions (Figure 4E). Interestingly, while MCP < 3 kDa showed relatively stronger Gal-3 inhibition in vitro, it did not accumulate in tumor tissue, suggesting that MCP’s potential anticancer effects are not driven solely by direct Gal-3 blockade in vivo. Larger fractions, like MCP > 30 kDa, appeared to remain longer in the tumor, reinforcing the concept that MCP’s activity likely arises from multiple, possibly indirect mechanisms rather than a single Gal-3 target.

Immunohistochemistry confirmed constitutive Gal-3 staining in SKOV-3 and MKN45 xenografts (Figure S5), indicating that low [^99^ ^m^Tc]­MCP tumor uptake is unlikely to be due to absence of tumor Gal-3. This assessment addresses expression only; activity and treatment-induced changes were not evaluated in this study.

MCP is a complex polysaccharide with diverse sources and a broad range of substructures.^63^ Its monomeric composition can vary significantly depending on the food source, extraction procedure, and modification strategy.^15^ In this study, we analyzed a commercial MCP and separated it into four fractions, revealing substantial differences in molecular weights and monosaccharide compositions. Such structural diversity is critical for understanding MCP’s biological effects and mechanisms of action.

One frequently investigated activity of MCP is its capacity to inhibit Gal-3, a protein involved in numerous physiological functions. Here, we observed that the MCP > 30 kDa and MCP < 3 kDa fractions contained higher levels of galactose, which has been reported as a potential Gal-3 inhibitor.^69^ Hemagglutination and Sepharose/Gal-3 assays showed that low-molecular-weight MCP fractions (notably MCP < 3 kDa) can bind and inhibit Gal-3 in vitro at high concentrations (25 mg/mL), albeit with relatively modest affinity. These findings suggest that MCP’s anticancer effects are not solely attributable to direct Gal-3 inhibition in vivo.

### 3.5 Galectin-3 Influences [^99m^Tc]­MCP Distribution in the Blood

To clarify Gal-3′s role in MCP distribution, we performed blood compartment and biodistribution studies in *Lgals3* ^+/+^ and *Lgals3* ^–/–^ mice. After IV injection of [^99m^Tc]­MCP, blood samples were collected for 60 min. In *Lgals3* ^+/+^ mice, [^99m^Tc]­MCP was mostly associated with plasma proteins (53.8% at 5 min, decreasing to 42% at 60 min), whereas approximately 24% persisted in blood cells (Figure 5A). In *Lgals3* ^–/–^ mice, the soluble fraction decreased more sharply, from 56.1% at 5 min to 7.6% at 60 min, indicating reduced plasma protein binding in the absence of Gal-3 (Figure 5B). Both groups exhibited predominant renal and hepatobiliary elimination, but kidney retention was higher in knockout mice (11.1% ID/g) than in wild-type (5.9%ID/g), suggesting that Gal-3 modulates MCP clearance rates (Figure 5C, Table 2).

**Figure 5:**
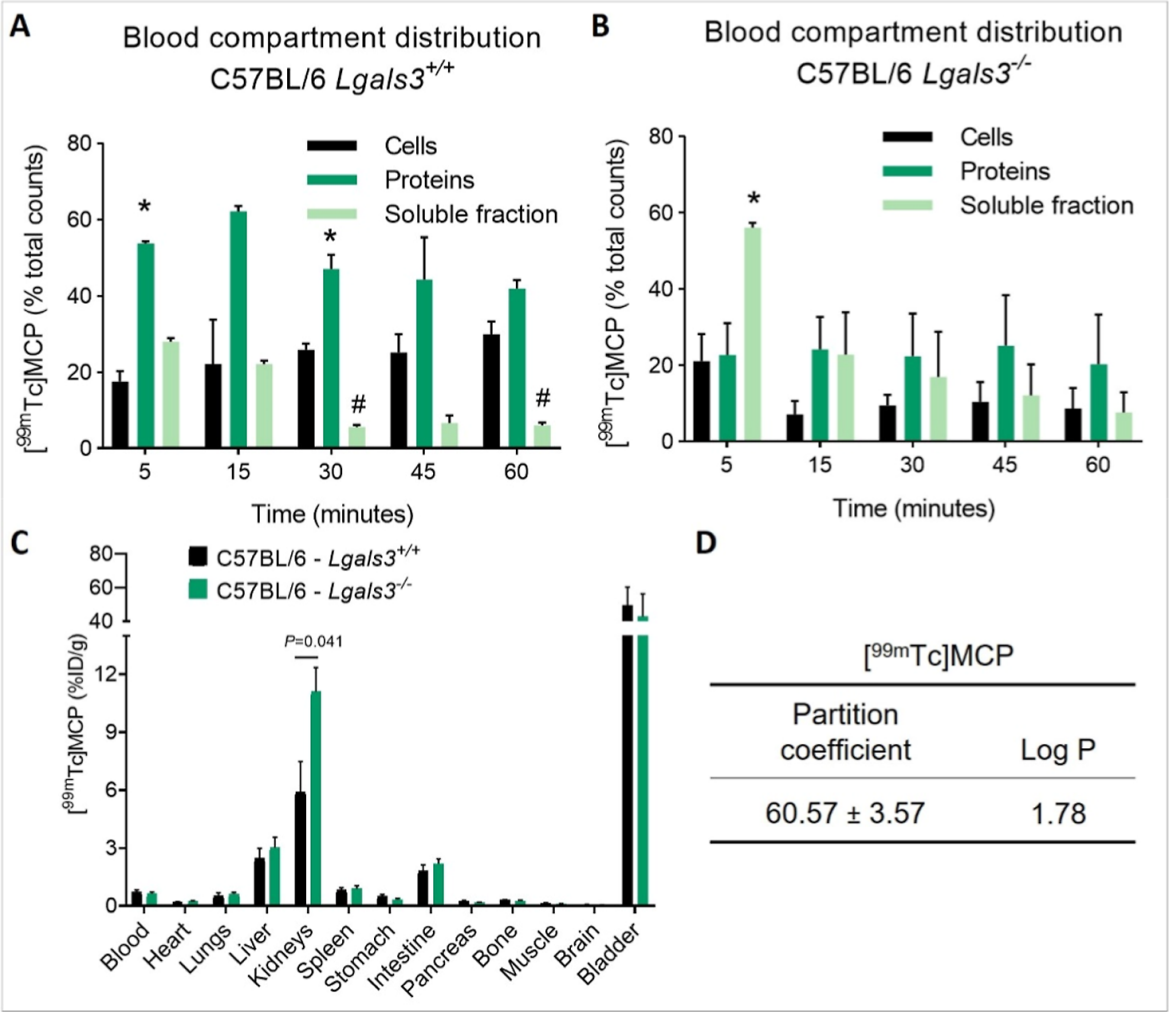
Galectin-3 influences the blood distribution and systemic clearance of [^99^ ^m^Tc]­MCP*in vivo*. Experiments performed using wild-type (C57BL/6*Lgals3* ^+/+^) and Gal-3 knockout (C57BL/6*Lgals3* ^–/–^) mice following intravenous injection of [^99^ ^m^Tc]­MCP. (A) Blood compartment distribution over time in *Lgals3* ^+/+^ mice, showing the percentage of total blood radioactivity associated with blood cells, plasma proteins (precipitated fraction), and the soluble plasma fraction. (B) Blood compartment distribution over time in *Lgals3* ^–/–^ mice (*p < 0.05 denoted by * for protein, # for soluble fraction comparison between genotypes at same time point). (C) Biodistribution of [^99^ ^m^Tc]­MCP at 1 h post-IV injection in *Lgals3* ^+/+^ and *Lgals3* ^–/–^ mice. Data show percentage of injected dose per gram (%ID/g). (D) Partition coefficient (log P) determination for [^99^ ^m^Tc]­MCP between *n*-octanol and saline (0.9% NaCl), indicating its relative lipophilicity/hydrophilicity. MCP = PectaSol-C (modified citrus pectin). Data are the mean ± SD of *n* = 5, *p < 0.05.

Although the lipophilicity (log *P* = 1.78) of [^99m^Tc]­MCP partly explains its plasma protein affinity, it does not fully account for the diminished binding in *Lgals3* ^–/–^ mice (Figure 5D). Previous studies have identified Gal-3 in blood plasma, urine, and various cell types,^70–75^ supporting its potential involvement in MCP’s distribution and clearance. In humans, its reference range in blood is approximately 17.8–22.2 ng/mL (Laboratory Corporation of America, Burlington, N.C., USA). Gal-3 is produced by endothelial cells, red blood cells, platelets, microparticles, and leukocytes, and it also exhibits prothrombotic properties in venous thrombosis.^76^ Our data indicate that MCP likely interacts with Gal-3 expressed on blood cells or in plasma, a process that may partially prolong MCP’s systemic circulation.

Given Gal-3′s known presence on blood cell membranes and in plasma, the differences in [^99m^Tc]­MCP distribution between *Lgals3* ^+/+^ and *Lgals3* ^–/–^ mice may derive from MCP’s interaction with Gal-3 binding sites. In *Lgals3* ^+/+^, this interaction appears to help retain MCP in the blood compartment, while in knockout mice, fewer potential binding sites lead to a faster decline of [^99m^Tc]­MCP in circulation. Despite Gal-3′s influence on blood distribution, biodistribution analyzes confirm that MCP undergoes both renal and hepatobiliary excretion in *Lgals3* ^+/+^ mice. However, in *Lgals3* ^–/–^ mice, the absence of Gal-3 correlates with a markedly more rapid elimination via the kidneys, suggesting that Gal-3 can prolong MCP’s systemic retention and potentially modulate its pharmacokinetic profile.

### 3.6 Galectin-3 Deficiency Accelerates [^99m^Tc]­MCP Elimination

We further examined how Gal-3 affects MCP’s pharmacokinetics. [^99m^Tc]­MCP (15 MBq, 100 μL) was injected intravenously into *Lgals3* ^+/+^ and *Lgals3* ^–/–^ mice, and serial blood samples were collected up to 1440 min postinjection. A two-phase decay model was applied.

The distribution half-life (*T*_1/2α_) did not differ significantly between wild-type (0.77 ± 0.06 min) and *Lgals3* ^–/–^ mice (2.40 ± 1.06 min), implying similar initial distribution rates. However, the elimination half-life (*T*_1/2β_) was significantly shorter in *Lgals3* ^–/–^ mice (303.53 ± 30.63 min) than in *Lgals3* ^+/+^ (502.89 ± 35.37 min), consistent with higher clearance (CL) in *Lgals3* ^–/–^ animals (2863.14 ± 129.40 μL/min) versus *Lgals3* ^+/+^ (1693.64 ± 368.39 μL/min). Volume of distribution (*V*_d_) showed no marked difference (Table 2).

These findings suggest that Gal-3 deficiency accelerates MCP clearance, possibly due to the presence of Gal-3 binding sites on plasma proteins or blood cells. Thus, while in vitro assays indicate that MCP can bind Gal-3, these results do not imply that in vivo antitumor activity is solely mediated by directly inhibiting Gal-3 in tumor tissue. Rather, Gal-3 appears to contribute to MCP’s systemic retention and clearance patterns. Consequently, these results support a model in which MCP’s potential anticancer activity arises from multiple systemic effects, rather than a single, direct blockade of tumor-associated Gal-3.

### 3.7 Clinical and Mechanistic Implications

Our quantitative imaging shows that orally delivered MCP attains <0.01% systemic bioavailability, explaining its lack of efficacy in graft models and challenging the premise of ongoing trials that rely solely on oral dosing for systemic cancers. Because intravenous MCP achieves therapeutically relevant exposure while oral MCP does not, future clinical studies should prioritize parenteral delivery or absorption-enhancing formulations when the treatment goal is extra-intestinal disease control. Finally, the fact that galectin-3 knockout mainly accelerates blood clearance indicates that MCP-galectin-3 interactions act systemically (e.g., in plasma or endothelium) rather than within the tumor microenvironment, supporting an indirect mechanism of antitumor action.

A growing body of work shows that extracellular galectin-3 can promote cancer progression at sites remote from the tumor parenchyma. Extracellular (soluble) galectin-3 binds complex *N*-glycans on VEGFR-2 and keeps the receptor clustered at the endothelial surface, thereby amplifying VEGF-A signaling and sustaining angiogenesis; correspondingly, genetic knock-down or systemic pharmacological blockade of galectin-3 slows the growth of xenografted tumors in nude or NOD/SCID mice.^23,76–80^

In the bloodstream, galectin-3 also binds the abundant carrier protein LGALS3BP/90 K, an interaction that stabilizes integrin-mediated adhesion and correlates with poor prognosis; neutralizing LGALS3BP limits vascularization and tumor size.^81^ By sequestering galectin-3 (and its binding partners) in the circulation, intravenously delivered MCP is likely to dampen these pro-angiogenic and pro-adhesive circuits, thereby retarding tumor growth without needing to accumulate in the tumor microenvironment or consistent with a mechanism that does not necessarily require adaptive immunity. This systemic “galectin-3 sponge” model reconciles our pharmacokinetic data with the robust antitumor effect observed in mice and provides a concrete, testable mechanism for future studies.

### 3.8 General Summary and Limitations

Overall, our results underscore MCP’s complexity and multifaceted actions. Using technetium-99m radiolabeling, we provide direct evidence of poor oral absorption, distinctive biodistribution, and Gal-3-influenced clearance. These findings suggest that MCP’s anticancer effects are likely systemic and indirect, rather than attributable to a singular Gal-3 blockade within tumors. Further studies are needed to elucidate how MCP’s molecular diversity and possible immunological or metabolic pathways contribute to its antitumor properties.

Despite in vitro evidence that MCP and other pectins can bind and inhibit Gal-3, no published study has conclusively demonstrated a direct in vivo MCP–Gal-3 interaction within the tumor microenvironment. In our work, faster MCP clearance in Lgals3–/– mice indicates a role for Gal-3 in systemic retention/distribution but does not confirm tumor-specific blockade. The absence of robust tumor uptake further argues against a mechanism requiring sustained intratumoral engagement. These gaps motivate targeted studies (e.g., in vivo colocalization imaging of MCP and Gal-3 or activity probes at tumor sites) before attributing efficacy to direct tumor-local Gal-3 inhibition.

## 4 Limitations

### 4.1 First, Immunodeficient Host Context and Tumor Microenvironment End Points (TME) Profiling

Efficacy was demonstrated in immunodeficient xenograft models (SKOV-3, MKN45) and in an immunocompetent syngeneic 4T1/BALB/c model; however, we did not perform immune profiling or depletion studies, so immune contributions cannot be mechanistically excluded. Tumor microenvironment end points were limited: beyond Gal-3 expression, we did not assess treatment-induced changes in angiogenesis, perfusion, proliferation/apoptosis, hypoxia, extracellular matrix, or immune infiltration. Given the host background and our proposed systemic mechanism, we now frame IV-MCP efficacy as consistent with circulation-level Gal-3 modulation. Future work will incorporate targeted TME analyzes (CD31, perfusion imaging, *K*_i_-67/cleaved caspase-3, Pimonidazole) and Gal-3-pathway readouts (plasma Gal-3/LGALS3BP, endothelial p-VEGFR2).

### 4.2 Second, Structural Attribution and GI Metabolites

Although we fractionated MCP by molecular weight and quantified monosaccharide composition, we did not assign specific glyco-epitopes or domains (e.g., RG-I side-chain galactans vs HG segments) to biological activity, nor did we identify in vivo GI degradation products. Our in vitro pH-stability data confirm that ^99m^Tc remains chelated to MCP in solution, but do not define chain length/branching of labeled species after GI transit. Accordingly, the oral data are interpreted as minimal systemic exposure to MCP-derived species under our regimen, without structural attribution of the active moieties.

### 4.3 Third, Radiochemistry/Plasma-Stability Window

IV plasma stability was sampled to 1 h. While in vitro plasma stability to 24 h and route-matched biodistribution/imaging support label retention within the study window, extended in vivo time points (e.g., 2–4 h with terminal cohorts) would further substantiate stability and exclude late dechelation artifacts.

### 4.4 Fourth, the Lack of Direct Interaction of MCP with Gal-3

The absence of robust tumor uptake of MCP further challenges the hypothesis that MCP’s antitumor effects rely on direct interactions with Gal-3 in the tumor microenvironment. Instead, the observed effects may be mediated by indirect systemic mechanisms or nonspecific binding to Gal-3 in extratumoral tissues or circulating compartments. Published data supporting Gal-3 inhibition by MCP primarily derive from in vitro studies or inferred from systemic observations, such as reduced metastasis or changes in immune modulation, rather than direct evidence of in vivo Gal-3 blockade in tumor tissues.

This gap underscores the need for more targeted studies employing advanced techniques, such as imaging-based colocalization of MCP and Gal-3 in vivo, or molecular probes designed to directly measure Gal-3 activity at tumor sites. Until such data become available, the in vivo role of Gal-3 inhibition by MCP remains speculative, and caution should be exercised in attributing its antitumor effects solely to this mechanism.

Our findings suggest that MCP’s antitumor activity is mediated primarily through indirect or systemic mechanisms rather than localized effects within the tumor microenvironment. Previous studies have proposed that MCP’s effects may involve immune system modulation, suppression of metastasis, or alterations in the extracellular matrix.^47,74,82^

### 4.5 Fifth, Model Selection and Translational Relevance

We studied two epithelial xenograft models—SKOV-3 (ovarian) and MKN45 (gastric)—and a 4T1 (breast) syngeneic tumor model. While route-dependent exposure and biodistribution are expected to be broadly informative for MCP, tumor uptake and antitumor effects may vary by histology, microenvironment, and dosing paradigm. The translational relevance of our findings to human patients must be interpreted with caution. Although mouse models are invaluable for mechanistic and proof-of-concept studies, they do not fully replicate the complexity of human cancers. Key differences in immune responses, metabolic pathways, and tumor heterogeneity between mice and humans could significantly impact the efficacy and pharmacokinetics of MCP.^75^ For example, human cancers typically exhibit more diverse stromal interactions and microenvironmental factors, which may influence MCP’s activity. Clinical studies are essential to validate our findings and to determine whether MCP demonstrates similar pharmacological effects in humans. Conclusions therefore apply to these models and conditions and should be generalized cautiously to other tumors or to humans. Planned studies in prostate (e.g., PC-3, LNCaP) and colon (e.g., HCT116, HT-29) models will test external validity, and clinical studies will be needed to establish human pharmacology and efficacy.

### 4.6 Sixth, Regimen Scope and Exposure–Response Design

We compared a single oral regimen (200 mg/kg) with a single IV regimen (10 mg/kg) given daily for 21 days; the study was not powered for per-animal exposure–response. Route-level PK showed IV dosing achieved higher systemic exposure (AUC_0–∞_) and coincided with antitumor activity, while tumor %ID/g was uniformly low—supporting efficacy that tracks systemic exposure rather than intratumoral accumulation. Because oral dosing used a higher mass, local GI burden and first-pass handling may explain observed biochemical changes and limit oral feasibility. Cross-study comparisons are hindered by heterogeneous reporting (% w/v in drinking water vs weight-based bolus); converting regimens to standardized units (daily/cumulative mg/kg) and running dose-ranging IV studies with matched per-animal PK—as well as oral PK/tox with osmolality/viscosity-matched controls and absorption-enhancing formulations—are needed to define a rigorous exposure–response and optimize route/schedule.

## 5 Conclusion

This study investigated the biological activity of modified citrus pectin (MCP, PectaSol-C) in an in vivo model, focusing on its absorption, biodistribution, and pharmacokinetics through both oral and intravenous administration, using molecular imaging techniques. We demonstrate that MCP (PectaSol-C) can be radiolabeled with technetium-99m, providing a practical tool to study its pharmacological behavior in vivo.

Our findings indicate that MCP exhibits significant anticancer activity when administered intravenously, but not orally, consistent with low absorption through the gastrointestinal tract. This has important implications for ongoing clinical trials using oral pectin formulations for cancer treatment, as limited systemic exposure may restrict therapeutic potential in the SKOV-3 and MKN45 xenograft models and the 4T1 syngeneic model evaluated here. At the same time, our data support the feasibility of [^99m^Tc]­MCP imaging and underscore the route dependence of MCP exposure; generalizability should be tested in additional tumor types and dosing paradigms. Collectively, these results suggest that alternative delivery strategies, such as intravenous administration or bioavailability-enhancing formulations, may be necessary to achieve meaningful clinical outcomes.

Furthermore, we evaluated the role of Gal-3 in MCP’s pharmacokinetics and biodistribution. While MCP demonstrated partial affinity for Gal-3 in vitro, our in vivo findings indicate that its anticancer effects are not exclusively dependent on Gal-3 inhibition, particularly within the tumor microenvironment. Instead, systemic mechanisms unrelated to direct tumor targeting may play a more prominent role in MCP’s activity, highlighting the need for further research into these pathways.

## Supplementary Material



## Notes

Supplementary figures and tables: Intravenous (IV) administration of MCP significantly reduced tumor growth and did not induce renal toxicity (Supplementary Figure 1). Biochemical analysis of animals treated with MCP (PectaSol-C, modified citrus pectin), both orally and intravenously (Supplementary Table 1). MCP can be radiolabeled with ^99^ ^m^Tc and remains stable in saline and blood plasma (Supplementary Figure 2). Representative whole-body μSPECT/CT imaging showing biodistribution of [^99^ ^m^Tc]­MCP (Supplementary Figure 3). Quantification of organs identified in SPECT images (% ID/g) (Supplementary Table 2). HPSEC profile and hemagglutination study controls (Supplementary Figure 4). Doses and activities by experiment (route, mass, activity) (Supplementary Table 3). Galectin-3 immunohistochemistry in SKOV-3 and MKN45 xenografts (Supplementary Figure 5) (PDF)
