# Supplementary material for: Investigation of Antitumor Activity of Modified Citrus Pectin: Oral and Intravenous Administration Assessed via Molecular Imaging
Source: Biomacromolecules. 2026 Mar 24;27(4):2449–65. doi: 10.1021/acs.biomac.5c00915 (PMC13080965; doi:10.1021/acs.biomac.5c00915)
Supplement: Supplementary file 1 [file bm5c00915_si_001.pdf]

Supporting information

Investigation of Antitumor Activity of Modified Citrus Pectin: Oral and Intravenous Administration Assessed via Molecular Imaging

Fábio Fernando Alves da Silva^[a]; Sofia Nascimento dos Santos^[b]; Lucas de Freitas Pedrosa^[c]; Vinicius Gonçalves Rodrigues^[d]; Jonathas Xavier Pereira^[d]; Jhonatas Pedrosa Marim Pereira^[a]; Dino Seigo Gushiken Junior^[e]; Thiécla Katiane Osvaldt Rosales^[c]; Luís Alberto Pereira Dias^[a]; Patrick Jack Spencer^[e]; João Paulo Fabi^[c]; Emerson Soares Bernardes^*[a].

(a) Instituto de Pesquisas Energéticas e Nucleares, Centro de Radiofarmácia, Comissão Nacional de Energia Nuclear, São Paulo, São Paulo, Brazil.

(b) School of Biomedical Engineering and Imaging Sciences, King's College London, London, SE1 7EH, UK.

(c) Department of Food Science and Experimental Nutrition, School of Pharmaceutical Sciences, University of São Paulo, São Paulo, São Paulo, Brazil.

(d) Instituto de Patologia Tropical e Saúde Pública (IPTSP), Universidade Federal de Goiás (UFG), Goiânia, Goiás, Brazil.

(e) Instituto de Pesquisas Energéticas e Nucleares, Centro de Biotecnologia, Comissão Nacional de Energia Nuclear, São Paulo, São Paulo, Brazil.

* Corresponding author: emerson.bernardes@gmail.com

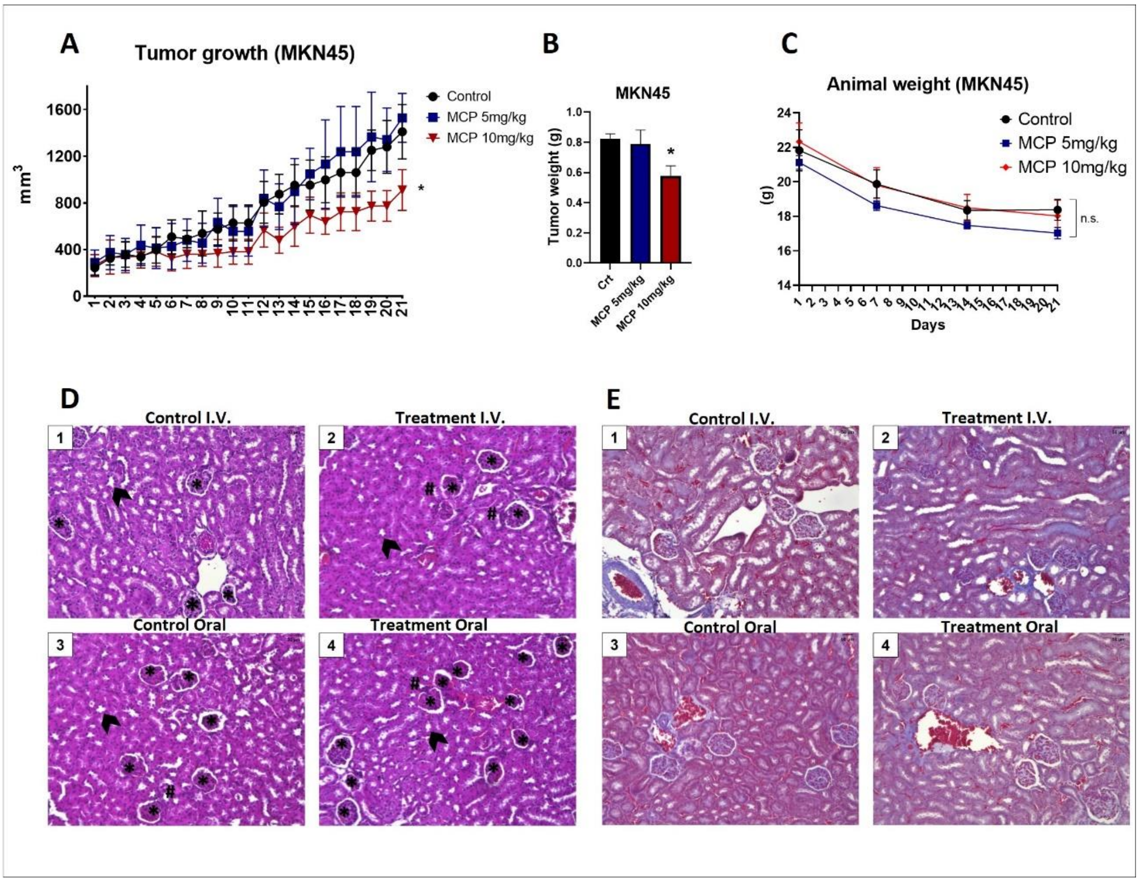

Supplementary Figure 1: Intravenous (IV) MCP significantly reduced tumor growth and did not cause renal toxicity. (A) Tumor volume (mm³) progression in BALB/c nude mice bearing subcutaneous MKN45 xenografts treated daily via intravenous injection with vehicle (Control, PBS), 5 mg/kg MCP, or 10 mg/kg MCP for 21 days. **(B)** Excised tumor weight (g) for MKN45 xenografts at the end of the study period for IV control and MCP treatment groups (5 mg/kg and 10 mg/kg). **(C)** Animal body weight (g) monitored throughout the 21-day IV treatment period for mice bearing MKN45 xenografts. **(D)** Representative photomicrographs of kidney histology (Hematoxylin and Eosin staining) after 21 days of treatment. Panels show cortical renal parenchyma from: (1) IV Control, (2) IV MCP (10 mg/kg), (3) Oral Control, (4) Oral MCP (200 mg/kg). Key structures indicated: renal glomeruli (*), proximal convoluted tubules (arrowheads), distal convoluted tubules (#). **(E)** Representative photomicrographs of kidney sections stained with Masson's trichrome after 21 days of treatment to assess extracellular matrix (ECM) deposition (collagen stains blue). Panels show cortical renal parenchyma from: (1) IV Control, (2) IV MCP (10 mg/kg), (3) Oral Control, (4) Oral MCP (200 mg/kg). MCP = PectaSol-C (modified citrus pectin). All histology images are representative of n=3 independent experiments. Magnification: 100×. For graphs A-C, data are shown as mean ± SD, n = 5 animals per group. Statistical significance vs. control indicated by * ($p < 0.05$); n.s. = not significant ($p > 0.05$). * Asterisks in panels D–E denote glomeruli; asterisks in graphs denote $p < 0.05$.

Supplementary Table 1: Biochemical analysis of animals treated with MCP (PectaSol-C, modified citrus pectin), both orally and intravenously.

	Healthy	Oral control	Oral MCP	IV control	IV MCP
**AST (TGO)**	40.53 ± 18.22 U/L	55.70 ± 1.10 U/L	84.45 ± 4.55 U/L *	34.32 ± 11.80 U/L	28.54 ± 11.80 U/L
**ALT**	54.03 ± 20.60 U/L	56.47 ± 5.45 U/L	42.11 ± 7.25 U/L	30.08 ± 10.12 U/L	42.27 ± 8.67 U/L
**ALP**	78.57 ± 31.20 U/L	58.53 ± 16.84 U/L	86.37 ± 9.88 U/L	80.21 ± 48.29 U/L	98.09 ± 0.84 U/L
**Urea**	43.08 ± 22.60 mg/dL	54.22 ± 18.40 mg/dL	49.30 ± 5.00 mg/dL	46.39 ± 2.89 mg/dL	52.70 ± 8.50 mg/dL
**Glucose**	95.10 ± 20.90 mg/dL	113.60 ± 47.10 mg/dL	134.10 ± 35.50 mg/dL	122.60 ± 27.40 mg/dL	86.90 ± 21.70 mg/dL
**Creatinine**	1.06 ± 0.20 mg/dL	0.85 ± 0.07 mg/dL	0.59 ± 0.05 mg/dL *	1.07 ± 0.30 mg/dL	1.01 ± 0.60 mg/dL

Data are mean ± SD; * $p < 0.05$ vs. corresponding route-matched control.

Abbreviations: ALT, alanine aminotransferase (also TGP); AST, aspartate aminotransferase (also TGO); ALP, alkaline phosphatase.

Units: Enzymes in U/L; metabolites in mg/dL unless noted.

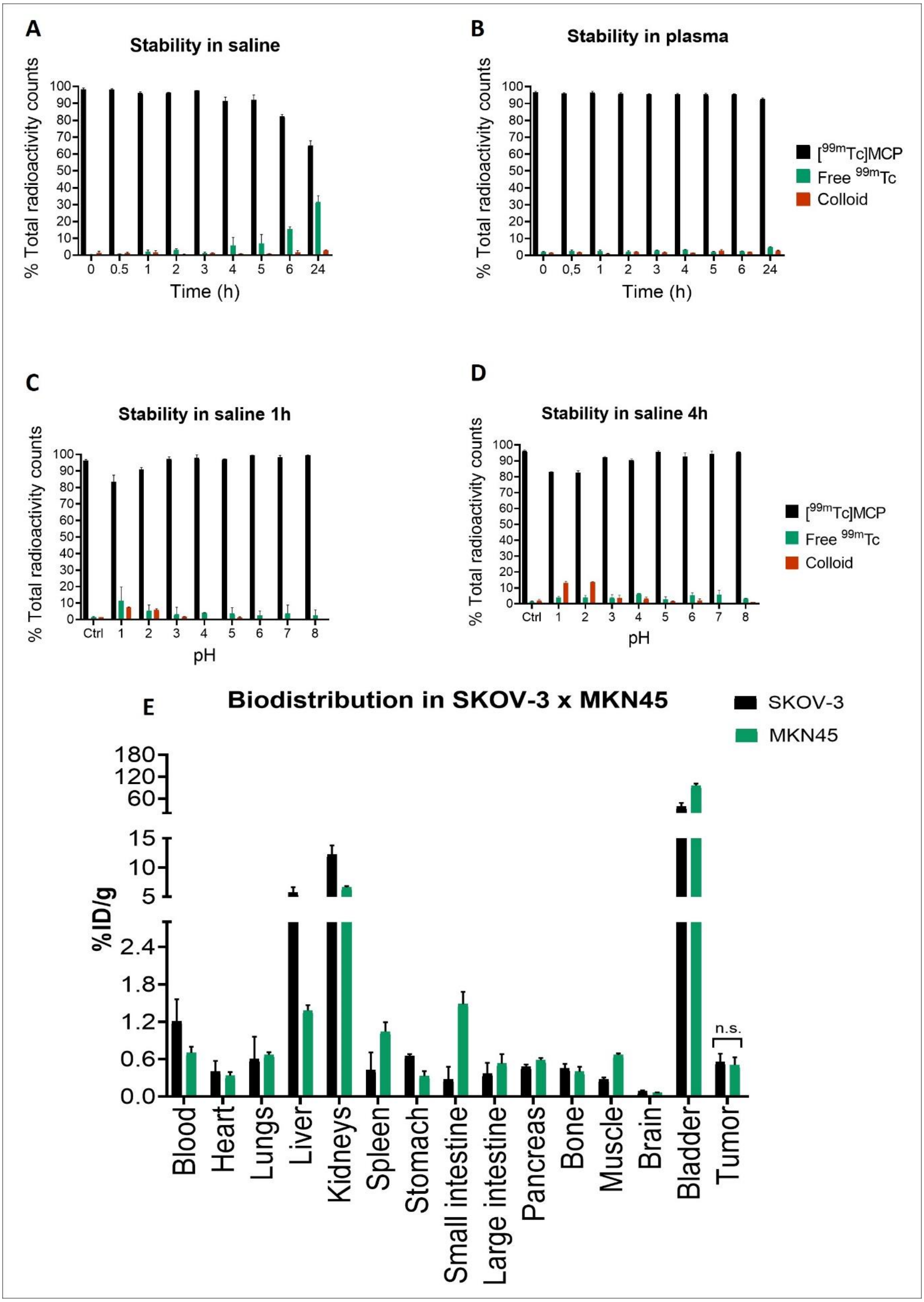

Supplementary Figure 2: MCP can be radiolabeled with ^{99m}Tc and remains stable in saline and blood plasma. (A) Stability of [^{99m}Tc]MCP (% total radioactivity) over 24 hours in saline (0.9% NaCl) at room temperature, assessed by ITLC-SG. Data show proportions of intact

[^{99m}Tc]MCP, free $^{99m}TcO_4^-$, and colloidal (reduced/hydrolyzed) ^{99m}Tc. **(B)** Stability of [^{99m}Tc]MCP (% total radioactivity) over 24 hours in mouse blood plasma at 37 °C, assessed by ITLC-SG. **(C)** Stability after 1 hour incubation in saline adjusted to pH 1–8, assessed by ITLC-SG. Ctrl = control (unadjusted pH). **(D)** Stability after 4 hours incubation in saline adjusted to pH 1–8, assessed by ITLC-SG. **(E)** Comparative biodistribution (% injected dose per gram, % ID/g) at 1 hour post-injection of [^{99m}Tc]MCP (10 MBq, intravenous) in BALB/c nude mice bearing SKOV-3 (black bars) or MKN45 (green bars) subcutaneous xenografts. Data show uptake in selected organs and tumors. MCP = PectaSol-C (modified citrus pectin). Data in A–E are presented as mean ± SD (n = 3–5 animals per group). n.s. = not significant ($p > 0.05$).

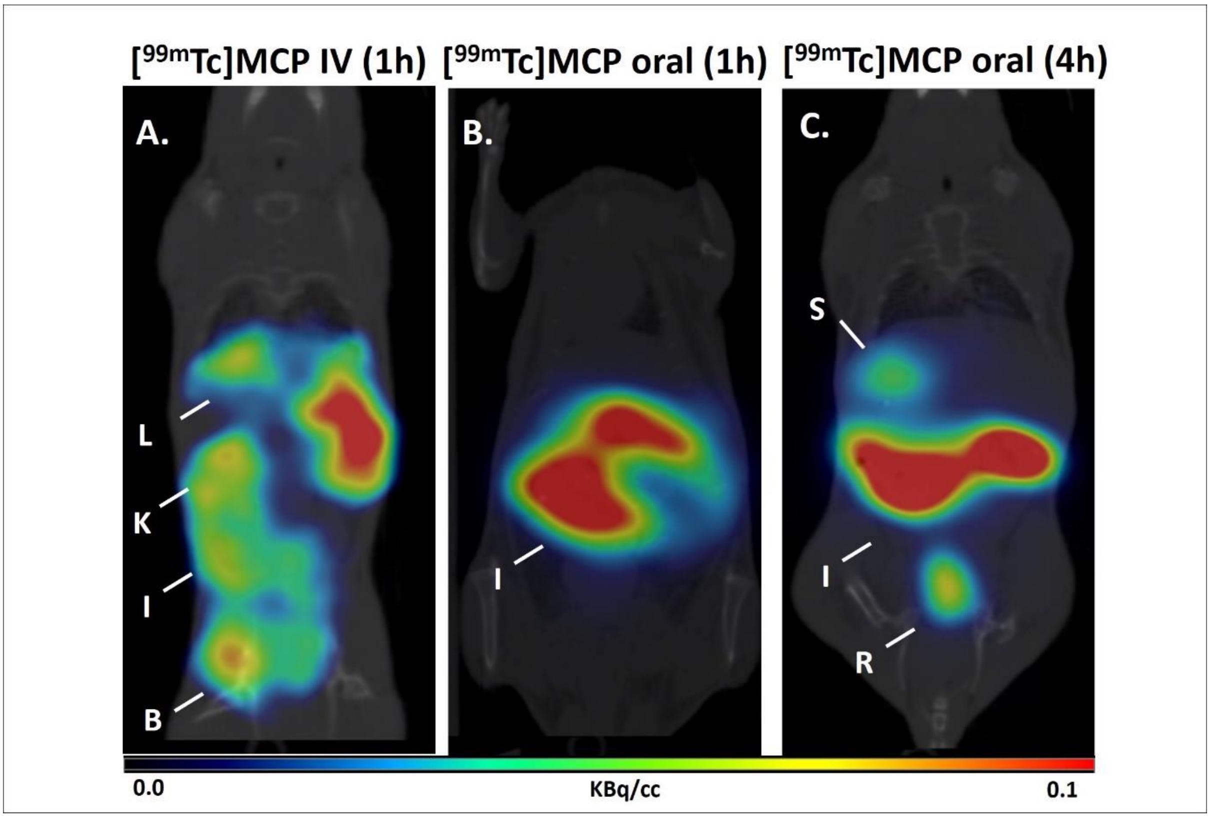

Supplementary Figure 3: Representative whole-body μSPECT/CT imaging showing biodistribution of [^{99m}Tc]MCP. Images acquired from BALB/c nude mice. Color scale indicates radioactivity concentration (kBq/cm³). **(A)** Image acquired 1 hour after intravenous administration of [^{99m}Tc]MCP (37 MBq), showing prominent signal in the kidneys (K), liver (L), and bladder (B), indicating renal and hepatobiliary clearance routes. **(B)** Image acquired 1 hour after oral administration of [^{99m}Tc]MCP (37 MBq), showing signal predominantly concentrated within the gastrointestinal tract (Intestine, I). **(C)** Image acquired 4 hours after oral administration of [^{99m}Tc]MCP (37 MBq), showing continued presence and distal movement of the signal within the gastrointestinal tract (intestine, I; rectum, R; stomach, S). MCP = PectaSol-C (modified citrus pectin). Images are representative of n = 3 independent experiments.

Supplementary Table 2: Quantification of organs identified in SPECT images (% ID/g). MCP = PectaSol-C (modified citrus pectin).

Organ	[^{99m}Tc]MCP IV, 1 h	[^{99m}Tc]MCP Oral, 1 h	[^{99m}Tc]MCP Oral, 4 h
**Small intestine**	ND	44.33 ± 8.76	19.75 ± 0.98
**Large intestine**	ND	43.09 ± 3.41	70.86 ± 4.79
**Rectum**	ND	ND	9.36 ± 0.97
**Bladder**	38.57 ± 8.33	ND	ND

ND: (not detected).

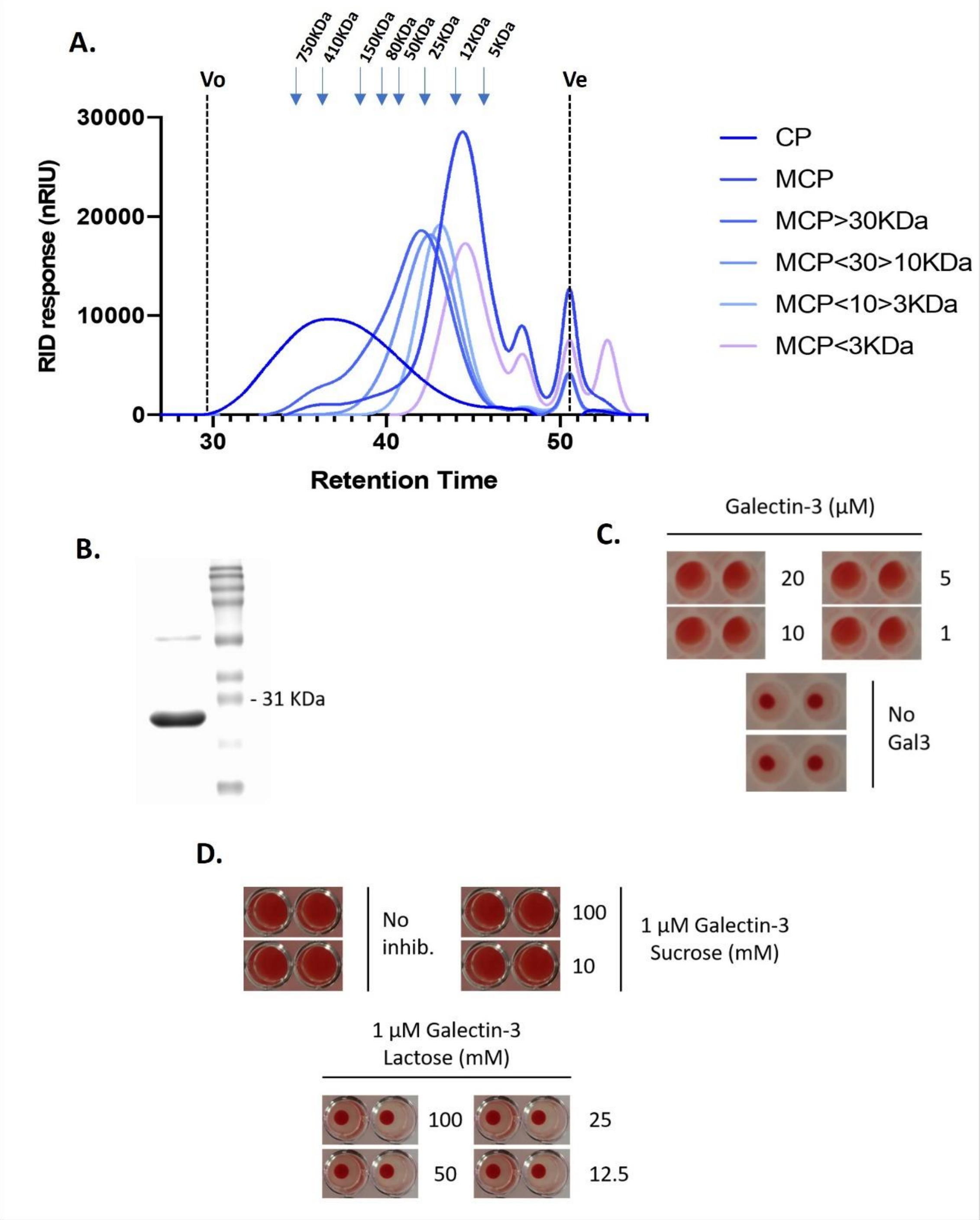

Supplementary Figure 4: HPSEC profile and hemagglutination study controls. (A) High-performance size-exclusion chromatography with a refractive index detector (HPSEC-RID) profiles showing molecular weight distribution of unfractionated Citrus Pectin (CP), Modified Citrus Pectin (MCP), and MCP fractions (MCP>30 kDa, MCP<30>10 kDa, MCP<10>3 kDa, MCP<3 kDa). Retention time is plotted against Refractive Index Detector (RID) response. Arrows indicate elution peaks of molecular weight standards (kDa). Vo = void volume, Ve = total volume. **(B)** SDS-PAGE analysis of purified recombinant galectin-3 under reducing conditions, stained with Coomassie blue. A prominent band is visible at approximately 31 kDa, confirming protein purity and expected molecular weight. **(C)** Control wells for the hemagglutination assay. Top row shows varying concentrations (1-20 µM) of galectin-3 in

hemagglutination assays; a button indicates no agglutination, while a diffuse mat/smear indicates agglutination. Bottom row shows no agglutination ('No Gal-3') in the absence of galectin-3. **(D)** Hemagglutination inhibition controls. Left panel ('No inhib. = no inhibitor') shows agglutination caused by 1 µM galectin-3. Middle panel shows lack of inhibition by sucrose (10, 100 mM). Right panel shows inhibition of galectin-3-mediated agglutination by lactose (12.5, 25, 50, 100 mM), a known galectin-3 inhibitor. MCP = PectaSol-C (modified citrus pectin). Assays are representative of n=3 independent experiments.

Supplementary Table 3: Doses and activities by experiment (route, mass, activity). MCP = PectaSol-C (modified citrus pectin).

**Experiment**	**Dose (MBq)**	**Injected MCP mass (mg/kg)**	**Injected volume (µL)**	**Rationale**
**Radiolabeling of MCP**	130	N/A	N/A	Better radiochemical efficiency
**Quality control**	20	N/A	N/A	Adequate counts
**Stability of [^{99m}Tc]MCP in saline, plasma and solutions of different pH**	20	N/A	N/A	Adequate counts
**Sepharose/Gal-3 column assay**	37	N/A	N/A	Adequate counts
**Stability of [^{99m}Tc]MCP *in vivo***	37	35.6	100	Higher activity to overcome low absorption; adequate tissue counts
**Biodistribution studies (IV)**	10	9.6	100	Adequate tissue counts IV; minimize burden
**Biodistribution studies (oral)**	37	35.6	100	Higher activity to overcome low absorption
**Kinetic studies (IV)**	15	14.4	100	Time-activity precision
**Kinetic studies (oral)**	37	35.6	100	Time-activity precision;

				higher activity to overcome low absorption
**Pharmacokinetic studies**	15	14.4	100	Time-activity precision
**µSPECT/CT imaging**	37	35.6	100	Scanner sensitivity/scan time balance
**Blood compartment distribution assay**	15	14.4	100	Time-activity precision
**Partition coefficient**	1	N/A	N/A	Adequate counts

N/A = No additions.

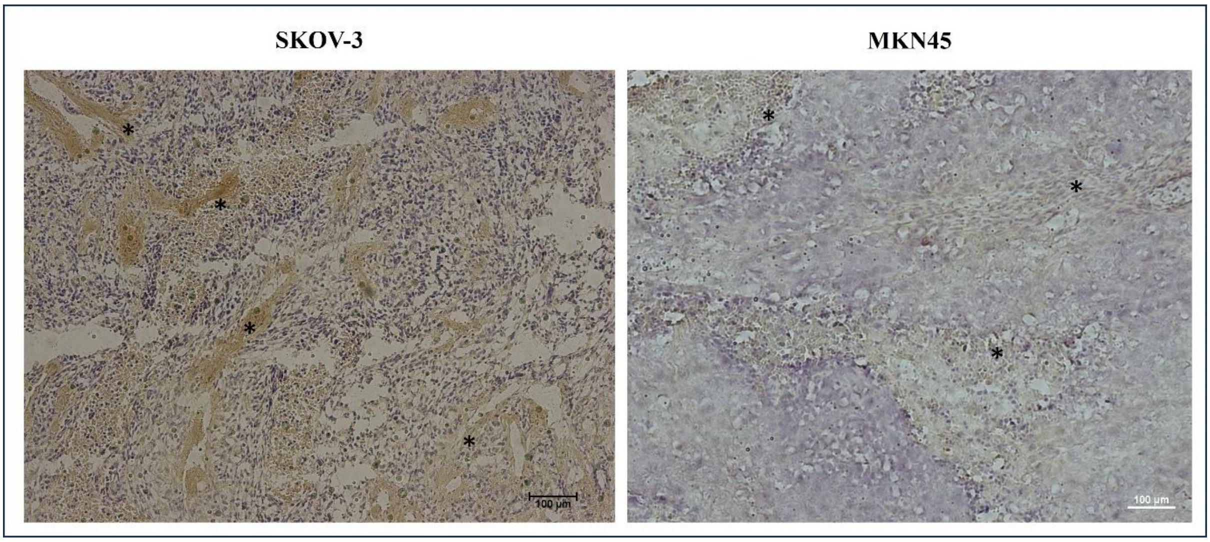

Supplementary Figure 5: Galectin-3 immunohistochemistry in SKOV-3 and MKN45 xenografts. Representative DAB staining (brown) with hematoxylin counterstain; scale bar = 100 µm; magnification 10×. Quantification method: Gal-3-positive area fraction/H-score over ≥3 fields per tumor. Data are descriptive and establish presence of Gal-3; they do not assess treatment-induced changes. Immunostaining assay of xenograft SKOV-3 and MKN45 cells for Gal-3 labeling. (*) regions of Gal-3 expression. Data are representative of three independent experiments.
